# The C-type lectin COLEC10 is predominantly produced by hepatic stellate cells and involved in the pathogenesis of liver fibrosis

**DOI:** 10.1038/s41419-023-06324-8

**Published:** 2023-11-30

**Authors:** Mengfan Zhang, Yang Jing, Wenze Xu, Xiaojing Shi, Wenguang Zhang, Pengfei Chen, Xiaocang Cao, Xinwei Han, Xuhua Duan, Jianzhuang Ren

**Affiliations:** 1 Department of Interventional Radiology, The First Affiliated Hospital of Zhengzhou University, Zhengzhou University, Zhengzhou, 450000 China; 2 Department of Hepato-Gastroenterology, Tianjin Medical University General Hospital, Tianjin Medical University, Tianjin, 300070 China

**Keywords:** Chronic inflammation, Diagnostic markers

## Abstract

Hepatic stellate cell is one of the major nonparenchymal cell types in liver. It has been proved the hepatic stellate cells are activated upon liver injury and produce excessive extracellular matrix to induce liver fibrosis. Single-cell RNA sequencing has been introduced to identify the subpopulations and function of hepatic stellate cells for its remarkable resolution of representation of single-cell transcriptome. According to the re-analysis of single-cell RNA sequencing data and pseudotime trajectory inference, we have found the C-type lectins including Colec10 and Colec11 are not produced by hepatocytes but predominantly produced by hepatic stellate cells, especially quiescent ones in the mice livers. In addition, the expression of Colec10 is decreased in the fibrotic livers of CCl4-challenged mice. COLEC10 is also mainly expressed in the hepatic stellate cells of human livers and the expression of COLEC10 is decreased with the progression of liver fibrosis. The bulk RNA sequencing data of the lentivirus transfected LX-2 cells indicates the function of COLEC10 is associated with inflammation, angiogenesis and extracellular matrix alteration. Surprisingly, the in vitro overexpression of COLEC10 in LX-2 cells promotes the mRNA expression of extracellular matrix components including COL1A1, COL1A2 and COL3A1 and the extracellular matrix degradation enzyme MMP2. To further investigate the role of COLEC10 in the pathogenesis of liver fibrosis, the serum concentration of COLEC10 in patients with chronic liver disease and healthy donors is measured. The serum concentration of COLEC10 is elevated in the patients with chronic liver disease compared to the healthy donors and positively correlated with serum concentration of the D-dimer but not the most of liver function markers. Altogether, we conclude that the C-type lectin COLEC10 is predominantly produced by the hepatic stellate cells and involved in the pathogenesis of liver fibrosis.

## Introduction

Hepatic stellate cell (HSC) is one of the major nonparenchymal cell type in liver. HSCs reside in the space of DISSE of liver sinusoid and maintain a quiescent state in healthy livers. Quiescent hepatic stellate cells (qHSCs) contain large droplets of Vitamin A ester in the cytoplasm and have a characteristic gene expression profile such as the expression of lecithin retinol acyltransferase (LRAT) and platelet derived growth factor (PDGF) receptor beta [1, 2]. Upon liver injury, hepatic stellate cells are activated and transdifferentiate to myofibroblast-like cells in respond to a serial of stimuli and cytokines. It has been demonstrated that the majority of myofibroblasts originates from hepatic stellate cells in the pathogenesis of liver injury [3]. The activation of HSC is driven by a various of endogenous and exogenous molecular stimuli and accompanied by profound changes of their transcriptome. A various of fibrogenic and proliferative cytokines including transforming growth factor beta (TGF-β) and PDGF can drive HSC activation. Endogenous metabolic factors including autophagy and endoplasmic reticulum stress also regulate activation and function of HSCs [2].

Activated hepatic stellate cells (aHSCs) acquire contractile property and have active ability of proliferation and producing extracellular matrix (ECM). The aHSCs reduce the expression of LRAT and characteristically express αSMA (alpha smooth muscle actin) and Collagen type 1 to support their contractility and ECM alteration ability [2]. In addition, it has been found that aHSCs are able to remodel the immune microenvironment via secreting various cytokines and immune molecules to regulate the function of immune cells. TGF-β secreted by the aHSCs not only activates qHSCs but also stimulates macrophages to be activated to a pro-fibrotic phenotype [4]. Interestingly, in vitro cultured primary rat HSCs reprogram their arginine-metabolizing enzymes with the decreased expression of inducible nitric oxide synthase and the increased expression of arginase-1 during activation, which is similar to the M2 polarization of macrophages [5]. The aHSCs promote the recruitment of macrophages via secreting C-C Motif Chemokine Ligand 2 (CCL2) [6]. In addition, human HSCs have been demonstrated to inhibit T-cell response via PD-L1/PD-1 pathway [7]. These data suggest HSCs also act as an essential immunoregulatory role in the liver microenvironment.

HSCs have been focused as a therapeutic target to alleviate and reverse liver fibrosis. Inhibition of HSC activation and /or elimination of aHSCs have been verified to reverse liver fibrosis in many preclinical studies. However, the mechanisms and specific markers of HSC activation are not fully elucidated which hinders the research and development of medication for liver fibrosis. With the development of single-cell RNA (scRNA) sequencing technique, the transcription profile of HSCs can be detected with a high resolution on each cell and the heterogeneity of HSCs is revealed clearly to help to discover the mechanism and specific markers of HSC activation. According to the results of scRNA sequencing of HSCs, the soluble guanylate cyclase has been found highly expressed in qHSCs and can be targeted to attenuate liver fibrosis with its activator [3]. In addition, specific markers of quiescent and activated HSCs are also clearly identified. For example, S100 calcium binding protein A6 (S100A6) has been discovered as a distinct marker of aHSCs [8]. The studies confirm that scRNA sequencing has a potent sensitivity and specificity to help to explore the subpopulations and specific functional genes of HSCs.

In this study, the scRNA sequencing data of hepatic stellate cells isolated from fibrotic mice livers were re-analyzed to screen highly expressed genes of qHSCs. Pseudotime trajectory inference was processed on the data to validate the gene expression trend during HSC activation. We have first demonstrated that the Colec10, that a member of C-type lectin family and considered to be produced by hepatocytes, is mainly produced by HSCs and hardly expressed in fully activated HSCs or hepatocytes, and have multiple functions including ECM production and alteration, immune response and cell interaction of vascular wall.

## Materials and methods

### Cell culture

LX-2 cells with STR verification were purchased from Procell Life Science & Technology Co., Ltd. (China). Cells were cultured in Dulbecco’s Modified Dulbecco’s Medium (DMEM) supplemented with 10% heat-inactivated fetal calf serum (Thermo Fisher Scientific), and antibiotics: 100 U/mL penicillin and 10 µg/mL streptomycin in an incubator containing 5% CO2 at 37 °C. The mycoplasma detection was performed regularly during the cell culture with a PCR kit (Beyotime, China). The culture cells used in the experiments were free of mycoplasma contamination.

### Animal study

The animal experiments were conducted in accordance with the ARRIVE guidelines (Animal Research: Reporting of In Vivo Experiments) for the care and use of laboratory animals, and the protocols were approved by the Zhengzhou University Animal Care and Use Committee (approval ZZU-LAC2022071902). Male C57BL/6 mice were housed under a standard SPF environment and fed chow and water ad libitum. The mouse liver fibrosis model was induced with 25% v/v carbon tetrachloride (CCl4) intraperitoneal injection (1 μL/g) twice a week for 2 and 4 weeks. Fifteen mice with the age of 6–8 weeks were allocated into the control group, 2 weeks group, and 4 weeks group. One mouse in the 4 weeks group failed to receive the full dosage of CCl4 and was excluded for the further analysis. The mice were euthanized humanely at 48 h after the last dose of CCl4. No blinding was used in the experiment.

### Clinical studies

The collection and study of human samples and clinical information were approved by the Ethics Committee of Tianjin General Hospital (Ethical NO. IRB2023-WZ-091). The liver samples were collected from 12 patients diagnosed as hepatitis and cirrhosis, respectively, via liver biopsy. The serum was collected from 13 healthy donors and a total of 56 patients diagnosed as chronic liver disease (CLD) with informed consent.

### Lenti-virus transfection

The COLEC10 overexpression LX-2 cells and control LX-2 cells were generated with pLV[Exp]-EGFP:T2A:Puro-EF1A > hCOLEC10[NM_006438.5] lentivirus (VectorBuilder, VB900010-2276yqs) and pLV[Exp]-EGFP/Puro-EF1A > ORF_stuffer lentivirus (VectorBuilder, VB010000-9389rbj). The virus transfection was performed following the manufacturer’s protocol. The cells were transfected with overexpression virus and control virus respectively. With puromycin incubation, transfected cells were expanded for further experiments.

### Adeno-associated virus transduction

The adeno-associated virus (AAV) carrying the coding sequence of mouse Colec10[NM_173422.3] and control AAV were generated by the Sangon Biotech (Shanghai, China). The serum type of AAV used in the experiment was AAV8. The virus titer of the virus pair was more than 1 × 10^12^ vg/ml before used. A total of 20 male C57/BL6 mice of 6–8 weeks were randomly allocated into two groups. 30 μl overexpression AAV or control AAV was diluted with PBS into 200 μl and then administered intravenously at the tail vein of each mouse. After 2 weeks, the AAV transduced mice were administered CCl4 intraperitoneally twice a week for 2 weeks. At 48 h after the last administration of CCl4, all the mice were humanely sacrificed. One mouse in the overexpression AAV group was died at the third administration of CCl4 and not include in the results of analysis.

### Quantitative real-time polymerase chain reaction

Gene expression levels were quantified by real-time reverse transcription polymerase chain reaction (RT-qPCR). Total mRNA was isolated from cells using Tri-reagent (Sigma Aldrich) according to the manufacturer’s protocol. Concentration of RNA was determined by Nano-Drop 2000c (Thermo Fisher Scientific). cDNA was synthesized from 0.5–2.5 µg RNA by HiScript 1st Strand cDNA Synthesis Kit (Vazyme, China). Gene expression was determined by primers with SybrGreen (Vazyme, China) by real-time polymerase chain reaction on the QuantStudio 3 system (Thermo Fisher Scientific). Relative gene expression was calculated via the 2-ΔΔCt method. The primers are shown in Table 1. All samples were measured in duplicate using RPS18(human) and Rps18(mouse) as housekeeping gene.

**Table 1:** Primers for RT-qPCR.

Species	Gene	Forward primer	Reverse primer
Human	RPS18	TGCGAGTACTCAACACCAACA	CTTCGGCCCACACCCTTAAT
	COL1A1	CCCCGAGGCTCTGAAGGT	GCAATACCAGGAGCACCATTG
	COL1A2	GGAGAGAGCGGTAACAAGGG	ACCAGGACTACCTCTCAGCC
	COL3A1	AAAGAGGATCTGAGGGCTCC	CAACACCACCACAGCAAGGA
	MMP2	CGTCGCCCATCATCAAGTTCC	TTCAGCACAAACAGGTTGCAG
	COLEC10	GGGAAAGCATGGCAAAGTGG	CCCTGATCTCCCATATCACCC
Mouse	Rps18	TGGGAAGTACAGCCAGGTTC	AGTGGTCTTGGTGTGCTGAC
	Acta2	AGAGCTACGAACTGCCTGAC	CGCTGACTCCATCCCAATGA
	Col1a1	GCAAGAGGCGAGAGAGGTTT	GGCACCAGTATCACCCTTGG
	Colec10	TAGTCGATCAGCTGCAGAAGT	CAGCTCTCCTTTCACTCCTTT
	Colec11	CTCCTTCGCCTAGTGTGCAT	CAAGAGCCAGGTCCCTCATC
	Il6	AGCCAGAGTCCTTCAGAGAGATA	TTGGTCCTTAGCCACTCCTTC
	Ifng	TGGCTGTTTCTGGCTGTTACT	TTCCACATCTATGCCACTTGA
	Tnf	CCCAAAGGGATGAGAAGTTCC	GCTCCTCCACTTGGTGGTT
	Il1b	AGCTCTCCACCTCAATGGAC	TTGTCGTTGCTTGGTTCTCCT
	Mrc1	TTCAGCTATTGGACGCGAGG	GAATCTGACACCCAGCGGAA
	Vegfa	CTCCACCATGCCAAGTGGTC	GTCCACCAGGGTCTCAATCG
	Mmp2	ATAACCTGGATGCCGTCGTG	CAGCCCAGCCAGTCTGATTT
	Tgfb1	GTGGAAATCAACGGGATCAGC	GTTGGTATCCAGGGCTCTCC

### Western blotting

Protein samples were prepared in lysis buffer (HEPES 25 mmol/L, KAc 150 mmol/L, EDTA pH 8.0 2 mmol/L, NP-40 0.1%, NaF 10 mmol/L, PMSF 50 mmol/L, aprotinin 1 µg/µL, pepstatin 1 µg/µL, leupeptin 1 µg/µL, DTT 1 mmol/L). Protein concentration was quantified by BCA protein assay (Beyotime, China) according to the manufacturer’s protocol using bovine serum albumin (BSA) to prepare a standard curve. Gel electrophoresis was performed with 10–20 µg protein using 4–15% gels (Beyotime, China), followed by transblotting to 0.2 µm nitrocellulose membrane (Amersham, USA). Protein band intensities were determined and detected with BeyoECL Star (Beyotime, China) using the Amersham Imager 680 system (GE). Primary antibodies used in the experiments, including anti-GAPDH (rabbit mAb, Beyotime, China), anti-GFP (mouse mAb, Beyotime, China), anti-COLEC10 (rabbit mAb, CUSABIO, China), anti-COL1A1 (rabbit pAb, ThermoFisher, USA), anti-αSMA (rabbit mAb, CST, USA), anti-GAPDH (mouse mAb, Beyotime, China) and β-Actin (Rabbit mAb, Cell Signaling, USA) were diluted 1:1000 in 1% BSA. Secondary antibodies used in the experiments including HRP-conjugated goat anti-rabbit IgG (Beyotime, China) and HRP-conjugated goat anti-mouse IgG (Beyotime, China) were diluted 1:1000 in 5% skimmed milk. The detailed information of antibody was listed in the supplementary file.

### Enzyme-linked immunosorbent assay (ELISA)

The blood samples were stored in EDTA tube and then centrifuged at 2–8 °C with 3000 rpm for 15 min and the supernatant were used for further analysis. The ELISA was performed following the manufacturer’s protocol (CUSABIO, China). The diluted serum samples and standard calibration samples were added into the pre-coated 96-well plates and then incubated at 37 °C for 1 h. After the solution was removed, the wells were washed with washing solution 30 s $\times$ 5 times. The enzyme conjugates were added to the wells with 50 μL per well except for the blank control wells and the plates were incubated at 37 °C for 30 min and then washed five times. The 100 μL TMB substrate solution was added into each well. After 10 min’ incubation, the reaction stop solution was added into each well and the plates were measured the optical density at 450 nm using SpectraMax i3x.

### Histology and immune staining

The paraffin embed samples were cut to 4 μm sections and performed with HE staining and Masson trichrome following the manufacturer’s protocol (Servicebio, China). Deparaffinized mouse liver sections were co-immunostained for COLEC10 (GeneTex, China) and HNF4a (Thermofisher, USA) with a two-step immunofluorescence kit (Beyotime, China). Deparaffinized human liver sections were co-immunostained for COLEC10 (CUSABIO, China) and COL1A1 (Beyotime, China) with a two-step immunohistochemistry kit (Beyotime, China) and co-stained for ALB (Beyotime, China) and COLEC10 (CUSABIO, China) with a two-step immunofluorescence kit (Beyotime, China). The quantification of the immunohistological staining was processed with ImageJ software with IHC profiler plugin. The detailed information of antibody was listed in the supplementary file.

### Bulk RNA sequencing and data analysis

The RNA sequencing of transfected LX-2 cells was performed and analyzed by Hangzhou KaiTai Biotechnology Co., Ltd, with Illumina Novaseq 6000 platform with PE150 mode. The public available bulk RNA sequencing data used in the manuscript were including GSE119606, GSE176042 and GSE193066 were collected from datasets and downloaded from GEO database [9–11]. The processing and analysis of public bulk RNA sequencing data were performed following a merged limma and edgeR workflow using R.

### Analysis of single-cell RNA sequencing data

The public available data of scRNA sequencing data and single-nuclear RNA sequencing data including GSE206409, GSE212039 and GSE212046 were collected from datasets and downloaded from GEO database [12]. The processing and analysis of data were followed the workflow of Seurat packages with R. The pseudotime trajectory inference were performed with Monocle3 package following the workflow of pseudotime cell trajectory inference.

### Statistical analysis

The Data were presented as mean ± standard deviation (mean ± SD) or mean ± standard error of mean (mean ± SEM). Statistical significance was analyzed by unpaired t-test or Mann–Whitney test (Wilcoxon test) between the two groups and by Kruskal-Wallis test between the multiple groups and the each group contains at least 3 independent samples. *p* < 0.05 was considered statistically significant (*: *p* < 0.05, **: *p* < 0.01, ***: *p* < 0.001, ****: *p* < 0.001, ns: *p* > 0.05). The analysis of data was performed using GraphPad Prism 9 (GraphPad Software) or R software.

## Results

### The landscape of hepatic stellate cells in the fibrotic mouse liver

**Fig. 1:**
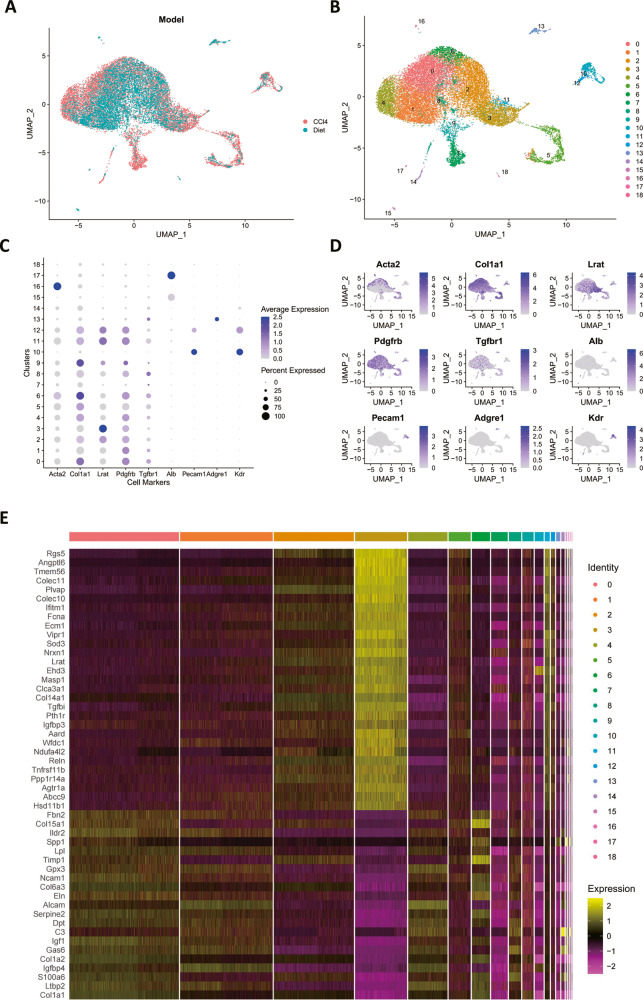
The gene expression profile of different hepatic stellate cell subpopulations. **A** The integration of scRNA-seq data of hepatic stellate cells from CCl4-induced and CDAA induced fibrotic mice livers. The data are collected from the GEO dataset GSE206409. **B** The clusters of hepatic stellate cells. **C** Dotplot of mRNA expression of cell marker genes. **D** Featureplot of mRNA expression of cell marker genes. **E** Heatmap of differentially expressed genes of quiescent hepatic stellate cells.

The RNA sequencing data acquired from isolated hepatic stellate cells of fibrotic mice livers induced by CCl4 and choline-deficient L-amino-defined diet (CDAA) respectively were integrated for further analysis and the sequenced cells were classified into 19 clusters with the uniform manifold approximation and projection (UMAP) dimension reduction method based on the expression profile of transcriptome of each cell (Fig. 1A, B). To identify the pure cell clusters of hepatic stellate cells, the mRNA expression of a few cell-specific genes was investigated and used for distinguishment of cell types. Lrat, Acta2, Col1a1, Tgfbr1, and Pdgfrb were used as markers of hepatics stellate cells. Alb was considered as hepatocyte-specific marker. Pecam1 and Kdr were selected as the markers of endothelial cells. Adgre1 was used as the Kupffer cell marker. The mRNA expression profile of the cell marker genes revealed that most of the clusters were hepatic stellate cells (Fig. 1C, D). Among all the clusters, cells of cluster 3 had the highest expression of Lrat and lowest expression of Acta2 and Col1a1. Therefore, we assumed that the hepatic stellate cells of cluster 3 predominantly consisted of qHSCs. Thereafter, the differentially expressed genes of cluster 3 compared to the rest clusters were calculated and the top 50 differentially expressed genes were represented as the heatmap to investigate the transcriptome of qHSCs (Fig. 1E).

### The pseudotime cell trajectory inference of hepatic stellate cell activation reveals Colec11 and Colec10 are highly expressed in quiescent hepatic stellate cells

**Fig. 2:**
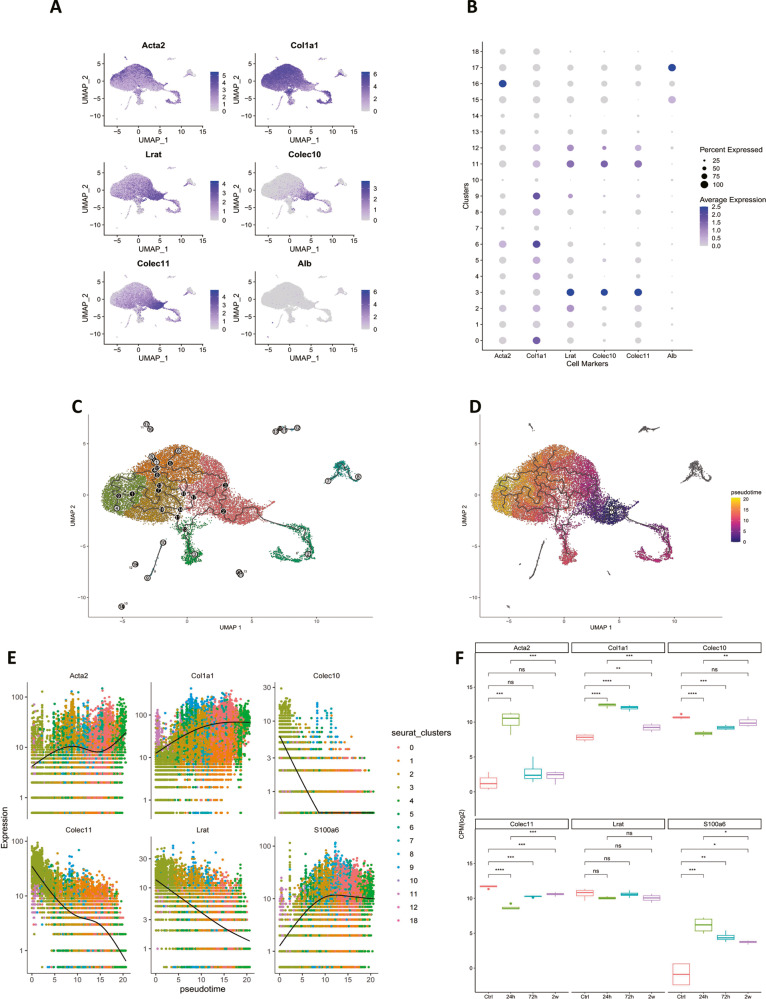
The C-type lectins including Colec10 and Colec11 are highly expressed in quiescent hepatic stellate cells. **A** Expression profile of Acta2, Col1a1, Lrat, Colec10, Colec11, and Alb in the whole cell population. **B** Dotplot of the expression of Acta2, Col1a1, Lrat, Colec10, Colec11, and Alb in each cluster. **C** Trajectories of hepatic stellate cell activation. **D** Pseudotime inference of hepatic stellate cell activation. **E** Expression trend of genes along with increased pseudotime. **F** Gene expression profile of isolated hepatic stellate cells from healthy mice livers (Ctrl) and CCl4 challenged mice livers (24 h, 72 h, and 2w). The data are collected from GEO dataset GSE176042.

Among the highly expressed genes of qHSCs, the C-type lectins including Colec11 and Colec10 draw our attention. The C-type lectin COLEC10 and COLEC11 were proved to be mainly produced by liver and kidney, respectively. In addition, the COLEC10 was deemed to be produced and secreted by the hepatocytes [13]. Colec11 has been identified as a conserved marker for zebrafish HSCs [14]. As far as we know, there has been no other evidence to demonstrate the HSCs produce the COLEC10. In the scRNA sequencing data, the featureplot and dotplot indicated the expression of Colec10 and Colec11 were mainly expressed in the Lrat-high and Acta2-low cells (Fig. 2A, B). The results suggested the COLEC10 and COLEC11 were highly expressed in the qHSCs. Since pseudotime trajectory inference was widely used to screen key molecules of cell differentiation in scRNA sequencing studies, we performed the pseudotime trajectory inference algorithm on the processed scRNA sequencing data of hepatic stellate cells. The qHSCs were selected as root cells and the cell trajectory plot and pseudotime plot were shown as Fig. 2D and Fig. 2E. The expression trend of genes demonstrated that the expression of S100a6, Acta2, and Col1a1 was gradually increased but the expression of Colec11, Colec10 and Lrat was gradually decreased as the pseudotime elapsed shown as Fig. 2E. The HSCs has been demonstrated to be self-reverted to an inactive phenotype without continuous stimulation of pro-fibrotic cues [15]. Bulk RNA sequencing data of isolated hepatic stellate cells from CCl4-challenged mice livers were re-analyzed. The results revealed that the expression of Colec11 and Colec10 in HSCs isolated from fibrotic mice livers was lower than that of HSCs isolated from healthy mice livers. Interestingly, the mRNA of Colec11 and Colec10 in HSCs was reciprocally expressed with the mRNA of Col1a1 and Acta2 in the in vitro cultured aHSCs, shown as Fig. 2G. The results indicated that the Colec11 and Colec10 were distinct high-expression genes of qHSCs.

### The C-type lectins including Colec11 and Colec10 were exclusively expressed in hepatic stellate cells and the gene expression of them was in the fibrotic liver of mouse models

**Fig. 3:**
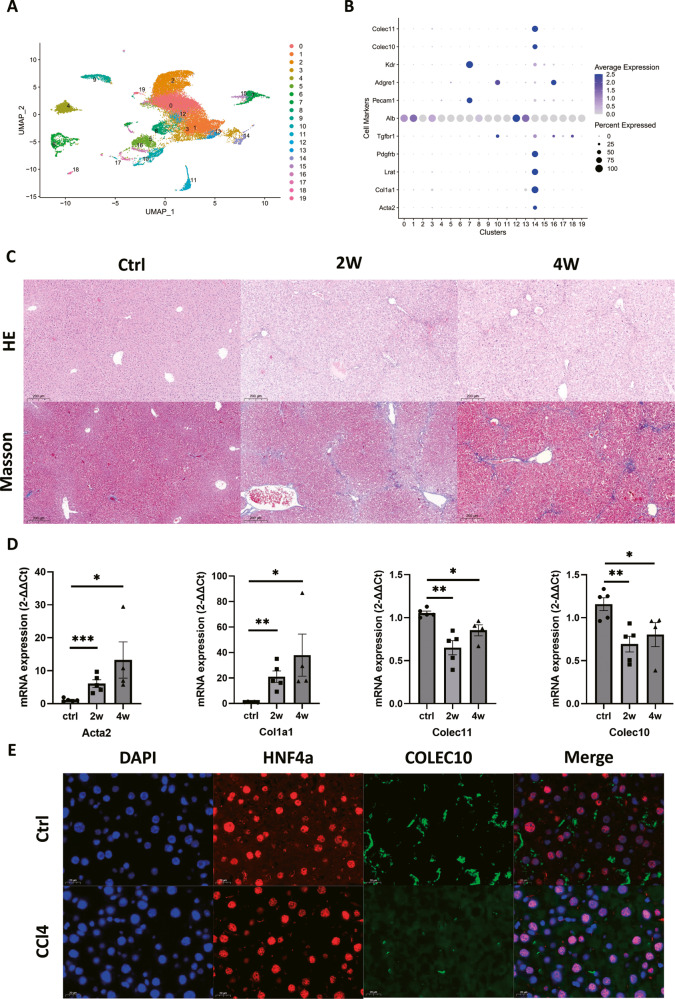
The C-type lectins including Colec11 and Colec10 are exclusively expressed in hepatic stellate cells and decreased in the fibrotic mice livers. **A** The landscape of cell clusters of isolated liver resident cells from CCL4-challenged mice livers. The data are collected from GEO dataset GSE212039. **B** The expression profile of cell marker genes in each cluster. **C** The HE staining and Masson trichrome staining of mice livers from mice treated with oil or CCl4 for 2 weeks or 4 weeks. Scale bar = 200 μm. **D** The RT-qPCR results of mRNA expression of mice livers treated with oil or CCl4 for 2 weeks or 4 weeks. **E** The immunofluorescence staining of HNF4a and COLEC10 of mice livers treated with oil or CCl4 for 2 weeks. Scale bar = 20 μm.

In the previous results, we have demonstrated that the Colec10 and Colec11 were highly expressed in the HSCs. Since the COLEC10 was deemed to be exclusively produced by hepatocytes, we were wondering the validity of the findings from scRNA sequencing data. The scRNA sequencing data from isolated liver resident cells of the fibrotic mice livers were collected and re-analyzed. The isolated liver cells were classified into 20 clusters based on the UMAP dimension reduction method (Fig. 3A). Thereafter, the marker genes of liver resident cells including hepatic stellate cells, hepatocytes, kupffer cells and endothelial cells were investigated in the scRNA sequencing data (Fig. 3B). The results demonstrated that the Colec10 and Colec11 were exclusively expressed in hepatic stellate cells. Since the Colec10 and Colec11 were highly expressed in the qHSCs, we assumed the two C-type lectins could be involved in the pathology of liver fibrosis. To testify the assumption, CCl4-induced liver fibrosis mouse models were generated. The HE staining and Masson trichrome staining revealed the scar tissue was observed in the mice livers challenged by 2 weeks and 4 weeks intraperitoneal injection of CCl4 (Fig. 3C). Furthermore, the mRNA expression of Acta2, Col1a1, Colec11 and Colec10 were quantified and the results demonstrated the activated hepatic stellate cell markers including Acta2 and Col1a1 were upregulated at 2 weeks group and 4 weeks group (Fig. 3D). The mRNA expression of Colec11 in fibrotic mice livers was decreased by about 40% at 2 weeks but was recovered a bit at 4 weeks. The mRNA expression of Colec10 was decreased by about 40% at 2 weeks in the fibrotic mice livers and remained the similar value at 4 weeks (Fig. 3D). The results implied the mRNA expression of Colec11 and Colec10 were downregulated in the hepatic stellate cells in the early phase of liver fibrosis. In addition, the livers of mice treated with oil or CCl4 for 2 weeks were strained for both of hepatocyte nuclear factor 4 alpha (HNF4a) and COLEC10. HNF4a is a specific hepatocyte nuclear receptor and can be used a lineage tracking markers of hepatocytes [16]. The staining results revealed that COLEC10 protein was predominantly located in the liver nonparenchymal stroma and its expression was reduced in the fibrotic mice livers (Fig. 3E). Accordingly, we speculated that the downregulation of Colec10 facilitated activation of hepatic stellate cells and accelerated liver fibrosis.

### The C-type lectins COLEC11 and COLEC10 are predominantly expressed in hepatic stellate cells in human livers

**Fig. 4:**
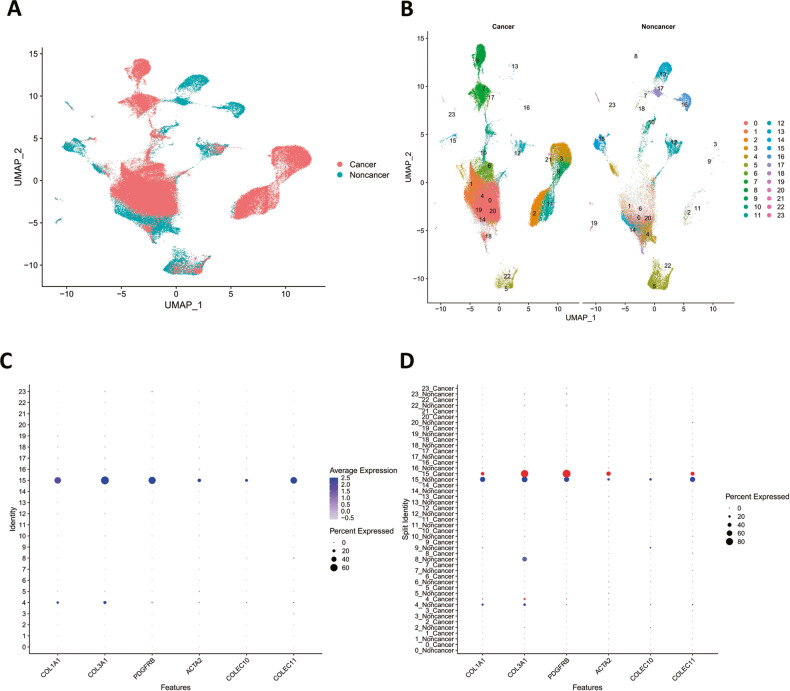
The landscape of single-nuclear RNA sequencing data from human liver cancer tissue and adjacent healthy liver tissue. **A** The integration overview of single-nuclear RNA sequencing data of human liver cancer tissues and adjacent healthy liver tissues. The data are collected from GSE212046. **B** The cell clusters of human liver cancer tissues and adjacent healthy liver tissues. **C** The gene expression amount and expressed cell ratio of hepatic stellate cell marker genes. **D** The gene expression amount and expressed cell ratio of hepatic stellate cell marker genes in human liver cancer tissues and adjacent healthy liver tissues.

In the previous results, we identified that the C-type lectins including Colec11 and Colec10 were mainly expressed in qHSCs in the mice livers. We were wondering whether COLEC11 and COLEC10 were highly expressed in human hepatic stellate cells. A human liver single-nuclear RNA sequencing dataset was collected and re-analyzed. The expression data from liver cancer tissues and adjacent healthy liver tissues were integrated for further analysis (Fig. 4A). The cells were classified into 24 clusters using UMAP dimension reduction algorithm (Fig. 4B). In the integrated data, the COLEC11 and COLEC10 were predominantly expressed in hepatic stellate cell clusters (Fig. 4C). Furthermore, the data were split into cancer cell data and noncancer cell data. We found the COLEC11 were expressed in both of cancer tissues and noncancer tissues, but the noncancer tissue had a higher expression of COLEC11. Interestingly, the COLEC10 were predominantly expressed in noncancer tissues (Fig. 4D). In addition, the hepatic stellate cells of liver cancer tissues had higher expression of activated markers including COL3A1 and ACTA2 than cells of noncancer tissues, which suggested the hepatic stellate cells of cancer tissues resembled aHSCs (Fig. 4D). The results verified the C-type lectins including COLEC11 and COLEC10 were hepatic stellate cells specific genes and the expression of COLEC10 was confined to qHSCs.

### The expression of COLEC10 was decreased during liver fibrosis progression

**Fig. 5:**
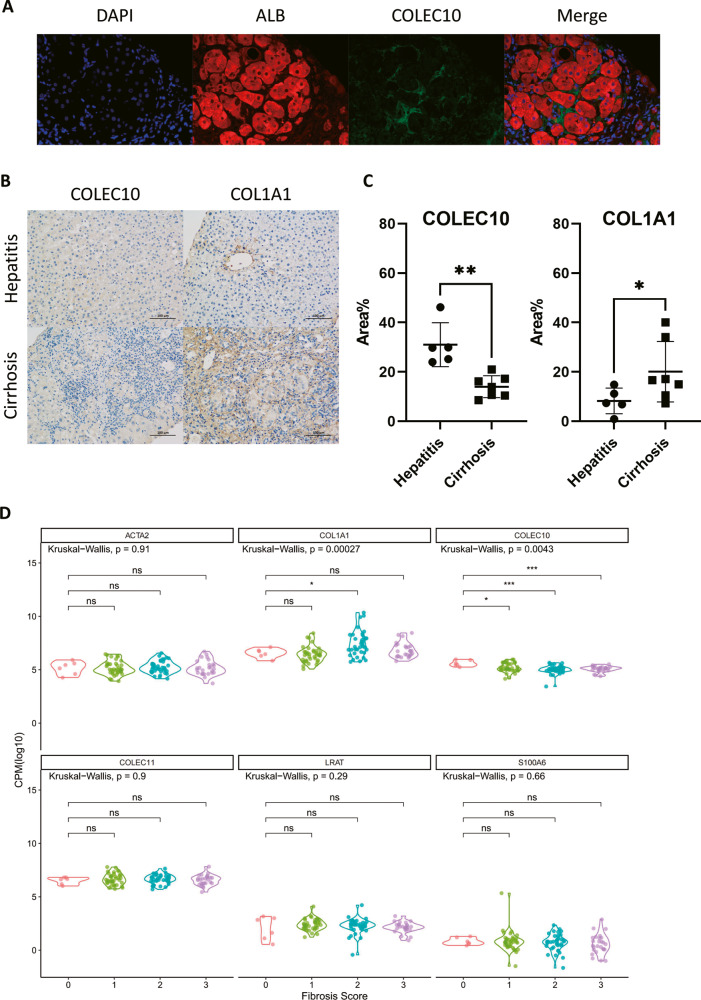
The expression of COLEC10 in human fibrotic livers. **A** The cirrhotic human liver section was co-stained for ALB and COLEC10. Magnification 400$\times$. **B** The human liver sections collected from patients with hepatitis or cirrhosis were stained for COLEC10 or COL1A1. Scale bar = 100 μm. **C** The positive area percentage of IHC images from patients with hepatitis or cirrhosis were quantified and compared. **D** The expression of interested genes in bulk RNA sequencing data of human liver samples from a cohort of patients diagnosed as liver fibrosis. The data are collected from GEO dataset GSE193066.

In the previous results, the COLEC10 was proven to be predominantly expressed in qHSCs. We were wondering whether the expression of COLEC10 was decreased in fibrotic and cirrhotic human livers. A human liver section collected from a cirrhotic patient was double stained for Albumin (ALB) and COLEC10 (Fig. 5A). The results revealed that the protein expression of COLEC10 was mainly located in nonparenchymal area adjacent to hepatocytes and was hardly observed in fibrotic area (Fig. 5B). We hypothesized that the expression of COLEC10 was negatively correlated with severity of liver fibrosis. Next, 12 cases of biopsy samples collected from patients diagnosed as hepatitis and cirrhosis were stained for COLEC10 and COL1A1. The IHC showed that the expression of COLEC10 was decreased and the expression of COL1A1 was increased in cirrhosis compared to hepatitis (Fig. 5C). The results were further verified with the comparison of quantified IHC images between hepatitis and cirrhosis (Fig. 5D). In addition, the bulk RNA sequencing data of liver biopsy samples from a cohort diagnosed with liver fibrosis were re-analyzed. The gene expression results revealed that the expression of LRAT, ACTA2, S100A6, and COLEC11 didn’t vary across different fibrosis scores, but the expression of COL1A1 and COLEC10 differed between samples with different fibrosis scores. The expression of COLEC10 was progressively decreased as the fibrosis scores of samples were increased. The results demonstrated the expression of COLEC10 was significantly negatively correlated with progression of liver fibrosis.

### The potential biological function of COLEC10 in human hepatic stellate cells revealed by RNA sequencing

**Fig. 6:**
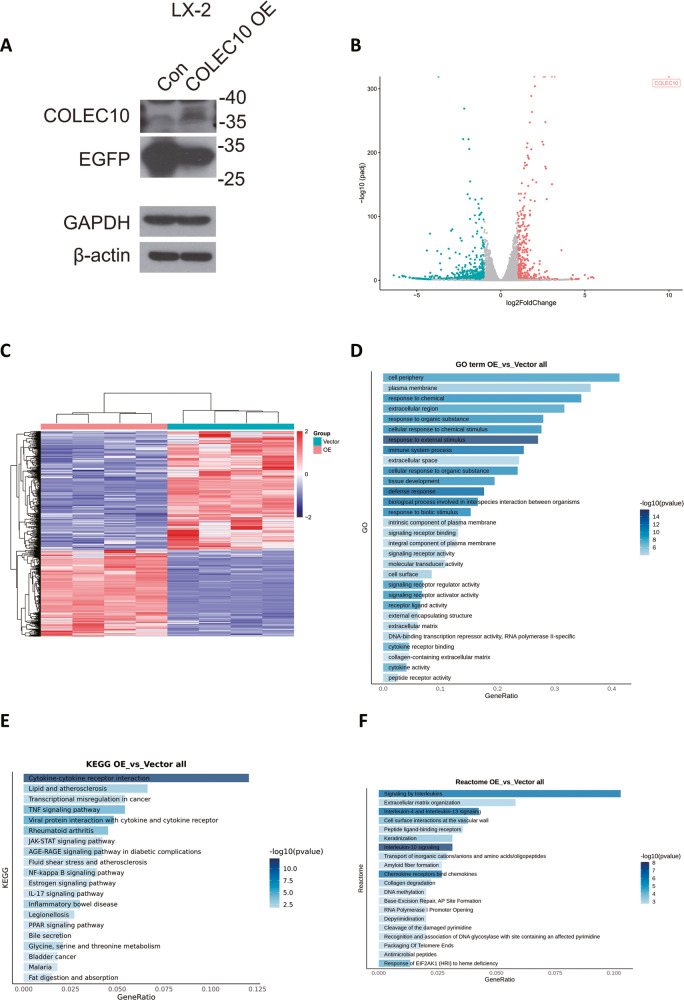
The effect of COLEC10 overexpression in LX-2 cells. **A** Protein expression of LX-2 cells transfected with control vector or COLEC10 over-expression vector. **B** The volcano plot of differentially expressed genes between vector transfected LX-2 cells and COLEC10 overexpression LX-2 cells in the RNA sequencing data. **C** The heatmap of scaled expression of differentially expressed genes in each sample of vector transfected LX-2 cells and COLEC10 overexpression LX-2 cells. **D** Gene Ontology (GO) classification of differentially expressed genes between samples. **E** The Kyoto Encyclopedia of Genes and Genomes (KEGG) classification of differentially expressed genes between samples. **F** The Reactome annotation pathway of differentially expressed genes.

To investigate the function of COLEC10 in the activation of hepatic stellate cells, the LX-2 cells, which were the immortalized cell line of human hepatic stellate cell, were transfected with COLEC10 overexpression lentiviral vector and control vector. The protein expression of COLEC10 in the transfected LX-2 cells were probed and the protein expression of COLEC10 was increased in the LX-2 cells transfected with COLEC10 overexpression lentivirus (Fig. 6A). The total mRNA of the transfected LX-2 cells was extracted and sequenced. The genes with the expression log2 fold change $>$ 1 and the adjust *p* value $<$ 0.5 were considered as differentially expressed genes for further analysis. A total of 423 upregulated genes (red dots) and 995 downregulated genes (green dots) were screened and shown as the volcano plot (Fig. 6B). The heatmap of scaled expression data of differentially expressed genes indicated the satisfied homogeneity of gene expression profile within each group of transfected LX-2 cells (Fig. 6C). The pathway enrichment analysis of differentially expressed genes were processed with GO annotation, KEGG annotation and Reactome annotation (Fig. 6C, D, E). Interestingly, the pathway analysis of Reactome database revealed the differentially expressed genes were annotated into the gene sets of Interleukin-4 and Interleukin-13 signaling and Cell surface interactions at the vascular wall (Fig. 6E). The interleukin-4 and interleukin-13 are well-documented type-2 immune cytokines and regulate tissue repair and fibrosis [17]. The dysfunctional sinusoidal endothelial cells are proved to be involved in the pathogenesis of liver fibrosis [18]. The results of pathway enrichment suggested that the function of COLEC10 was closely related to the interaction of hepatic stellate cells with immune cells and sinusoidal endothelial cells.

### The hepatic stellate cell expressed COLEC10 is associated with inflammatory regulation and extracellular matrix alteration

**Fig. 7:**
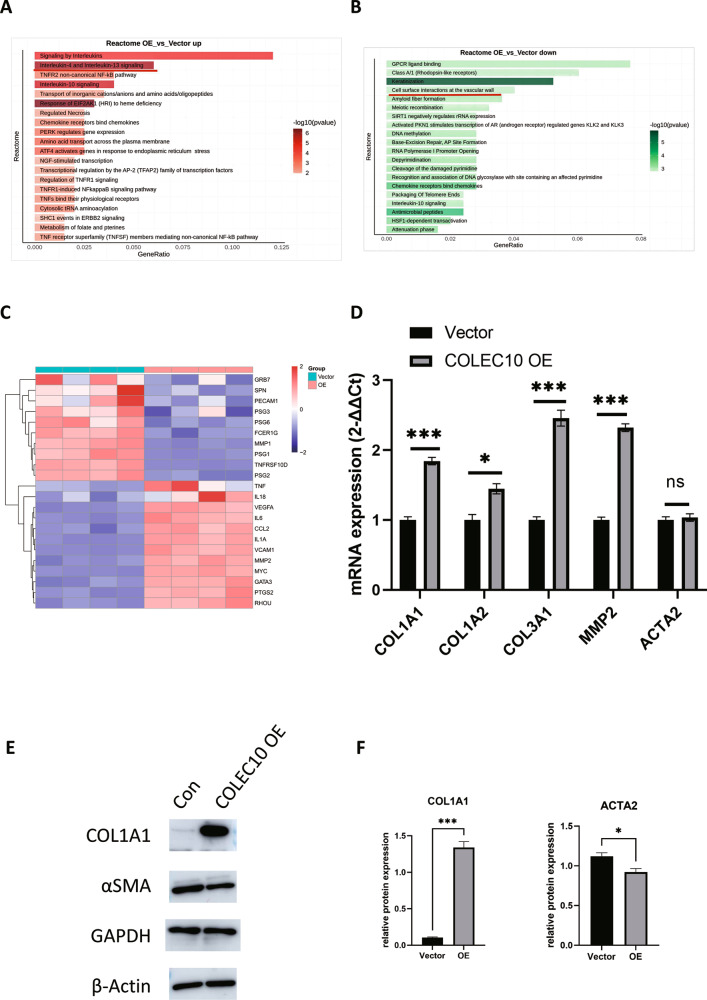
The immune regulation and extracellular matrix alteration associated genes are increased in the COLEC10 over-expression LX-2 cells. **A** The Reactome pathway analysis of upregulated genes in the COLEC10 over-expression LX-2 cells. **B** The Reactome pathway analysis of downregulated genes in the COLEC10 over-expression LX-2 cells. **C** The heatmap of scaled expression of genes in the interleukin-4 and interleukin-13 signaling pathway and the cell surface interactions at the vascular wall pathway between the COLEC10 over-expression LX-2 cells and the vector control LX-2 cells. **D** The mRNA expression of COL1A1, COL1A2, COL3A1, MMP2 and ACTA2 in the COLEC10 over-expression LX-2 cells and the vector control LX-2 cells. The GAPDH and β-Actin were used as loading control. **E** The protein expression of COL1A1 and ACTA2 in the COLEC10 over-expression LX-2 cells and the vector control LX-2 cells. **F** The quantificiation of protein expression of COL1A1 and ACTA2 in the COLEC10 over-expression LX-2 cells and the vector control LX-2 cells.

To further annotate the functions of differentially expressed genes in the COLEC10 over-expression LX-2 cells, the upregulated and downregulated genes were performed with Reactome pathway analysis respectively. The results indicated the upregulated genes were associated with the interleukin-4 and interleukin-13 signaling pathway and the downregulated genes were associated with the cell surface interactions at the vascular wall pathway, shown as Fig. 7A, B. The heatmap of scaled expression of the differentially expressed genes enriched in the two pathways was shown as Fig. 7C. A few of the downregulated genes including PSG1, PSG2, PSG3, and PSG6 in the COLEC10 over-expression LX-2 cells are members of the carcinoembryonic antigen family and capable to activate TGF-β in ECM [19]. The inflammatory cytokines including tumor necrosis factor (TNF), interleukin-6 (IL6) and interleukin-1a (IL1A) and vascular endothelial growth factor a (VEGFA), vascular cell adhesion molecule 1 (VCAM1), matrix metallopeptidase 2 (MMP2) and CCL2 were upregulated in COLEC10 over-expression LX-2 cells, shown as Fig. 7C. The function of the upregulated genes is associated with inflammation, angiogenesis and ECM alteration. The results revealed the COLEC10 is involved in the pathogenesis of liver fibrosis. Since the scRNA-seq results and bulk RNA-seq results revealed that the mRNA of COL1A1 and COLEC10 was reciprocally expressed in the HSCs, we were wondering the effect of COLEC10 on the collagen production in the LX-2 cells. The mRNA expression of COL1A1, COL1A2 and the COL3A1 was probed by RT-qPCR. Surprisingly, the expression of the collagen producing mRNA was increased in the COLEC10 over-expression LX-2 cells, shown as Fig. 7D. Meanwhile, the mRNA expression of ECM degrading enzyme MMP2 was increased but the mRNA expression of ACTA2 didn’t change in the COLEC10 over-expression LX-2 cells. For the further validation of the qPCR results, the protein expression of COL1A1 and ACTA2 in the LX-2 cells were probed by western blotting and quantified and the results demonstrated that the COLEC10 increased by about 10-fold protein expression of COL1A1 in LX-2 cells and decreased by about 10% of the ACTA2 protein expression, shown as Fig. 7E, F. The results demonstrated the COLEC10 increased collagen production of LX-2 cells and suggested the effect of COLEC10 was resulted from both of transcriptional and post-transcriptional mechanism. Taken together, we concluded that the COLEC10 produced by HSCs promoted tissue repair with multifunction including ECM production and alteration, angiogenesis, and inflammatory regulation.

### Serum COLEC10 is increased in the patients with chronic liver disease and positively correlated with D-dimer concentration

**Fig. 8:**
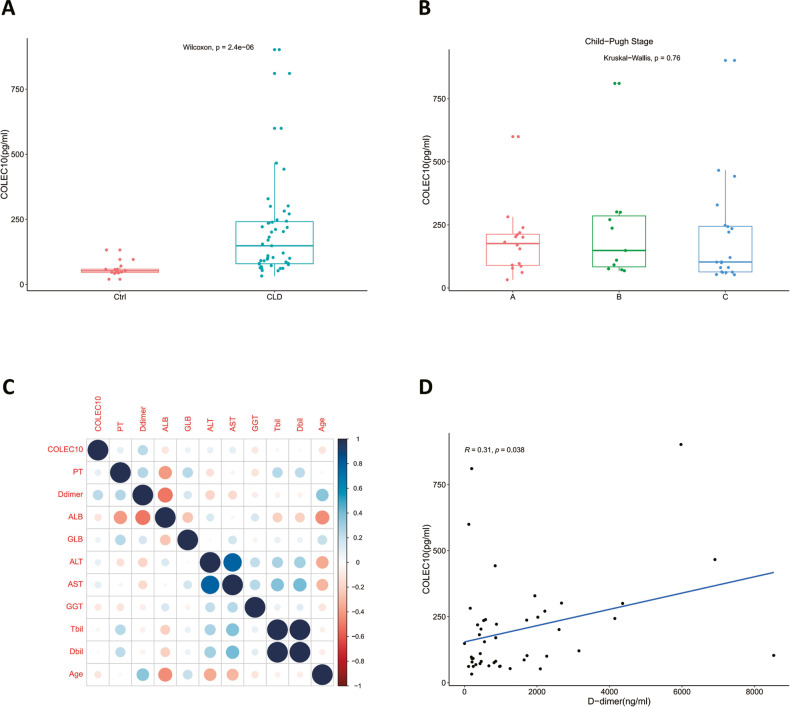
The serum COLEC10 is increased in patients with chronic liver disease. **A** The comparison of serum COLEC10 concentration between healthy donors and patients with chronic liver diseases (CLD). **B** The serum concentration of COLE10 across different Child-Pugh stages of CLD patients. **C** The correlation plot of COLEC10 with other serum markers and clinical features of patients with CLD. **D** The linear regression of COLEC10 to D-dimer in the patients with CLD.

COLEC10 was considered as a soluble C-type lectin mainly produced by liver. In our previous results, it has been shown the mRNA expression of the Colec10 in the fibrotic livers was decreased in both of mice models and biopsy samples of patients. The in vitro experiments demonstrated the COLEC10 promoted tissue repair. We were wondering the association between concentration of serum COLEC10 and chronic liver diseases (CLD). Serum of a consecutive cohort of 56 patients diagnosed with CLD and 13 healthy donors was collected and the COLEC10 concentration was quantified via ELISA. The results exhibited the serum concentration of COLEC10 was increased in the patients with CLD (Fig. 8A). However, the concentration of COLEC10 didn’t differ between different Child-Pugh stages, which indicated the production of COLEC10 might not be determined by the function of hepatocytes (Fig. 8B). Thereafter, we analyzed the correlation between the serum COLEC10 concentration and the clinical serum markers and clinical characteristics in the patients with CLD. The correlation matrix of the concentration of clinical serum markers including prothrombin time (PT), D-dimer, albumin (ALB), globulin (GLB), alanine aminotransferase (ALT), aspartate aminotransferase (AST), gamma glutamyl transferase (GGT), total bilirubin (Tbil), direct bilirubin (Dbil) and clinical features including age of patients with CLD suggested that the serum concentration of COLEC10 was positively correlated with serum concentration of D-dimer, shown as Fig. 8C. Then we performed the univariate linear regression of COLEC10 to D-dimer with Pearson correlation test and the results revealed the correlation coefficient was 0.31 with statistical significance (*p* = 0.038), shown as Fig. 8D. D-dimer is a product via the process of fibrinolysis and upregulated in the patients with vascular thrombosis including thrombosis of portal vein. The portal venous thrombosis is a common pathological change in the patients complicated by cirrhosis [20]. The serum COLEC10 was upregulated in the patients with CLD and positively correlated with serum D-dimer concentration, which suggested the serum concentration of COLEC10 was closely correlated with the microvascular pathological changes of chronic liver disease and independent of the function of hepatocytes.

### In vivo over-expression of COLEC10 contributes to ECM alteration and angiogenesis of fibrotic mice livers

**Fig. 9:**
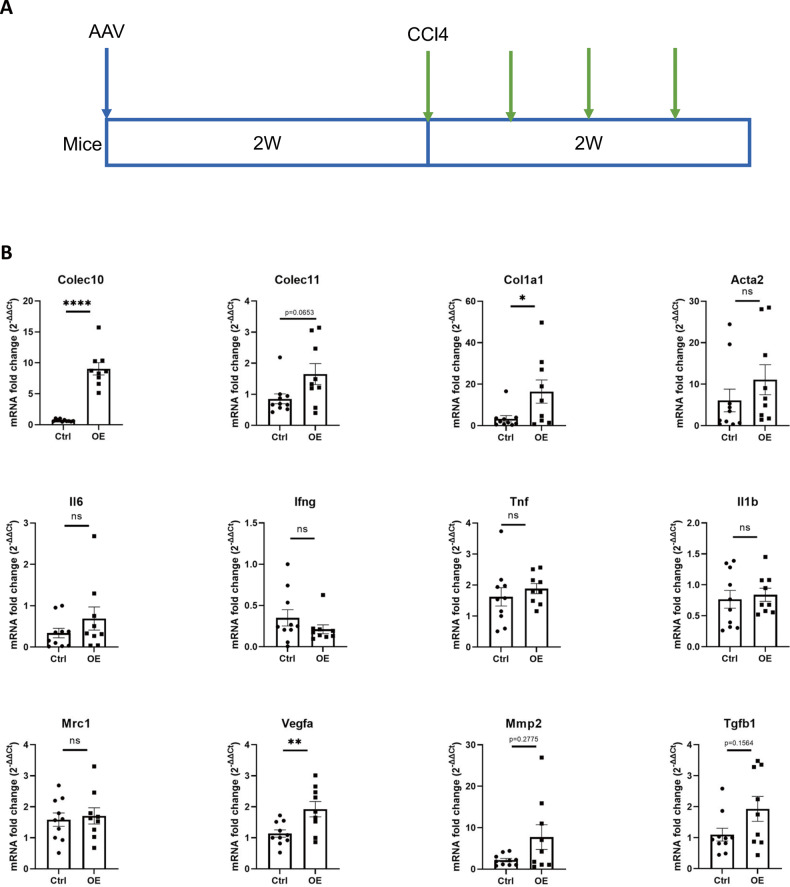
AAV transduced COLEC10 overexpression contributes to ECM alteration and angiogenesis in CCl4 challenged mice livers. **A** Illustration of AAV transduction and CCl4 administration of mice models. **B** mRNA expression of genes of mice livers in AAV control group (Ctrl) and AAV overexpression group (OE).

In our previous results, we have found that the collagen production was increased in COLEC10 overexpression LX-2 cells. Therefore, we were wondering if the effect of COLEC10 remains similar as that in fibrotic mice models in vivo. The liver-specific serum type of AAV was chosen to overexpress COLEC10 in CCl4-induced liver fibrosis mice models. The AAV carrying coding sequence of Colec10 and control AAV were intravenously injected into mice followed by 2-week intraperitoneally administration of CCl4, shown as Fig. 9A. To investigate the in vivo effect of COLEC10, mRNA expression of a few genes that involved in the pathogenesis of liver fibrosis was measured, shown as Fig. 9B. As shown in the results, the mRNA expression of Colec10 was increased by about 10-fold in the mice livers in overexpression group indicating the AAV transduction successfully promoted the expression of Colec10 in the mice livers. The mRNA expression of Colec11 of overexpression group was about 2-fold than that of control group, though difference of was not statistically significant. The mRNA expression of Col1a1 was increased by almost 20-fold in the overexpression which demonstrated the COLEC10 promoted the collagen production in vivo. However, the mRNA expression of Acta2 in both groups was at a similar level. The mRNA expression of pro-inflammatory genes including Il6, Ifng, Tnf and Il1b was also detected in the two groups but none of them varied between both groups. In addition, the genes associated with wound repair, including Mrc1, Vegfa, Mmp2 and Tgfb, were also probed in the two groups. The mRNA expression of Vegfa was significantly increased by 1-fold in the overexpression group. Although the average expression of Mmp2 and Tgfb was higher in the overexpression group than that in control group but the difference between two groups was not statistically significant. The mRNA expression of M2 macrophage marker Mrc1 didn’t show much difference between the two groups. In conclusion, the COLEC10 overexpression in vivo promoted the mRNA expression of Col1a1 and Vegfa, which suggested that the COLEC10 is associated with the ECM alteration and angiogenesis in the pathogenesis of liver fibrosis.

## Discussion

With the re-analysis of scRNA-seq data and bulk RNA sequencing data from liver samples and isolated hepatic stellate cells, we first demonstrate the COLEC10, a member of the C-type lectin family considered to be mainly produced in hepatocytes, is mainly expressed in the qHSCs and reciprocally expressed with Col1a1 and Acta2. The images of immunofluorescence staining on mice livers and human livers reveal that the COLEC10 is localized in nonparenchymal area of liver. The mRNA expression of COLEC10 is decreased in the fibrotic mice livers and the fibrotic human livers. The overexpression of COLEC10 in immortalized human hepatic stellate cells, LX-2 cells, modulates the expression of genes associated with type-2 immune response and cell interactions at vascular wall and promotes the expression of Collagens. The results suggest the function of COLEC10 is closely correlated with tissue repair mechanism in liver diseases. In addition, we have analyzed the serum concentration of COLEC10 in patients with CLD and healthy people. The serum concentration of COLEC10 is elevated in CLD patients and positively correlated with serum concentration of D-dimer, indicating the potential role of COLEC10 in microvascular pathological changes of diseased livers. To our knowledge, our study demonstrates the COLEC10 is produced by qHSCs and involved in the pathogenesis of liver fibrosis for the first time.

CLD induced with various etiology, including toxic injury, viral infection, autoimmune diseases, metabolic and genetic diseases, possibly evolve into liver fibrosis. Liver fibrosis is considered as a reversive state but has a risk to progress to cirrhosis and lead to liver cancers [1, 21]. HSCs are the major nonparenchymal cells in liver, which accounts for about 10% of all resident liver cells [2]. HSCs remain a quiescent state but are activated and transdifferentiated to myofibroblasts upon liver injury. The distinct morphology of qHSCs is the storage of retinyl esters in their cytoplasmic lipid droplets. The LRAT, which catalyzes the esterification of all-trans-retinol into all-trans-retinyl ester, is highly expressed in qHSCs and widely used as a marker to monitor the activation of HSCs [22]. In response to the liver injury, aHSCs produce and secret collagens, especially collagen type 1 and collagen type 3 to form the ECM, and characteristically express matrix metalloproteinases including MMP2 and the tissue inhibitor of metalloproteinases including TIMP2 to alter the structure and ingredients of ECM to facilitate tissue repair. In addition to its role in ECM alteration, HSCs secret a variety of cytokines including TGF-β and PDGF, chemokines including CCL2, and other inflammatory regulatory molecules to modulate the function of HSCs themselves and the immune cells in the microenvironment of damaged liver [6, 23]. Interestingly, the senescent HSCs enhance their ability to secret pro-inflammatory molecules to induce M1 polarization, which considered as a protective mechanism to limit the progression of liver fibrosis [24]. The current studies demonstrate the significant role of HSCs in the pathogenesis of chronic liver disease.

We first demonstrate the COLEC10 is predominantly expressed in qHSCs and hardly expressed in the aHSCs or hepatocytes. The COLEC10 is a member of the C-type lectin family containing more than 1000 proteins which are defined by having one or more calcium dependent characteristic carbohydrate recognition domains. Among them, the function of soluble C-type lectins varies. For example, soluble C-type lectins can function as growth factors, opsonins, antimicrobial proteins and ingredients of the ECM, and they regulate many essential processes such as development, respiration, coagulation, angiogenesis and inflammation [25]. The Collectin-10, encoded by the gene COLEC10, is considered to be produced in the hepatocytes of the liver [13, 26]. A recent study has shown that the proteins of COLEC10 and COLEC11 form a heteromeric complex and can be detected in the endo-/exocrine secretory tissue and mucosa, but the mRNA of COLEC10 is highly expressed in the liver and placenta, which indicates the production of COLEC10 is mainly in the adults’ liver [27]. In a recent study, COLEC11 is identified as a conserved marker of zebrafish HSCs [14]. According to the results of scRNA-seq, the COLEC10 is predominantly expressed in the qHSCs. The result is further verified by the RNA-seq data from transfected LX-2 cells and TGF-β treated primary human HSCs that the COLEC10 is barely expressed in LX-cells and TGF-β stimulation inhibits the expression of COLEC10 in primary human HSCs, shown as Supplementary Fig. S1. These data indicate that the COLEC10 is not expressed in the aHSCs. Considering the expression of COLEC10 is reciprocally correlated with ACTA2, we conclude that the COLEC10 is predominantly expressed in the qHSCs. The stiff matrix has been demonstrated to promote the expression of ACTA2 in LX-2 cells and the activation of HSCs [28, 29]. Interestingly, the expression of COLEC10 is decreased in the fibrotic livers and mainly detected in the non-fibrotic area which is revealed by the immunofluorescence staining of fibrotic mice livers and human livers, shown as Fig. 3E and Fig. 5A. Therefore, we assume the increased stiffness in the fibrotic liver tissues further inhibits the expression of COLEC10 in qHSCs in addition to the pro-fibrotic cues. However, to our knowledge, the COLEC10 has not been reported to be produced by HSCs elsewhere. The inaccurate localization of COLEC10 may be an obstacle to explore its function.

In view of the current research about the function of COLEC10, little is known about its role in the liver diseases. The mutation of COLEC10 is reported to be associated with mutations of MASP-1/3 and COLEC10 to lead to a rare autosomal recessive genetic disorder named of the Malpuech, Michels, Mingarelli, Carnevale (3MC) syndrome [30]. The 3MC syndrome is characterized by a spectrum of developmental abnormalities may present more than one of the following symptoms including high-arched eyebrows, cleft lip/palate, hearing loss, short stature, umbilical hernias/omphalocele, and urogenital abnormalities [30]. The pathogenesis of the 3MC syndrome is speculated to be associated with the deficient activation of complement, coagulation, and kallikrein–kinin systems and abnormal cell migration ability during embryonic development [31–33]. Apart from the development disorder, overexpression of COLEC10 in hepatocellular carcinoma cells induces endoplasmic reticulum stress and inhibits the progression of liver cancer [34]. Interestingly, the mining of the RNA-seq data from The Cancer Genome Atlas (TCGA) indicates the high expression of COLEC10 in the liver cancer tissue predicts a better prognosis of the patients [34, 35]. Considering the COLEC10 is mainly produced in the HSCs, the real function of COLEC10 in HSCs remains unelucidated. LX-2 cells are immortalized human hepatic stellate cells resembling the function and molecular features of primary activated HSCs and are highly transfectable [23]. The mRNA expression of COLEC10 in LX-2 cells is quite low, shown as Supplementary Fig. S1. To comprehensively investigate the function of COLEC10 in HSCs, the bulk RNA sequencing is used to screen the differentially expressed genes. The differentially expressed genes in the COLEC10 overexpression LX-2 cells are closely correlated with inflammation, angiogenesis, and ECM alteration via upregulating the mRNA expression of TNF, IL18, IL6, IL1A, CCL2, MMP2, VEGFA and VCAM1. In addition, we have demonstrated the COLEC10 promoted the mRNA expression of collagens including COL1A1, COL1A2 and COL3A1 and collagenase MMP2. The enhanced collagen production of LX-2 cells by COLEC10 overexpression is also verified by the western blotting. The collagens are the main components of ECM to server as the scaffold of regenerative cells in the process of wound repair. MMP2 can be activated by collagens and degrades the collagens and is essential for the remodeling of ECM [36]. Our results suggest the COLEC10 in hepatic stellate cells may act an essential role in the repair mechanism upon liver injury. Interestingly, the mRNA expression of ACTA2 wasn’t affected by COLEC10 but the protein expression of ACTA2 was decreased by 10% by COLEC10. The data indicate the COLEC10 may hinder the contractile ability of aHSCs via decreasing the expression of ACTA2. Altogether, our results indicate that the COLEC10 is a pluripotent C-type lectin to promote tissue repair.

The heteromeric complexes of COLEC11 and COLEC10 have been found in the human serum and able to mediate complement activation interacting with mannan-binding lectin-associated serine proteases (MASP) [37]. The clinical significance has been investigated in a few cohort studies. For example, high serum concentration of COLEC10 and COLEC11 is associated with mortality of kidney transplant recipients [38]. The serum concentration of COLEC10 in patients with acute liver failure and cirrhosis is higher than healthy people [39]. However, few studies highlight the clinical significance of COLEC10 in the patients with chronic liver disease. Our analysis of serum samples demonstrates the serum concentration of COLEC10 is upregulated in the patients with CLD. In addition, we find that the serum concentration of COLEC10 does not differ between patients with different Child-Pugh stage and is not correlated with most of the clinical markers of liver function, which suggests that the serum concentration of COLEC10 is indispensable of the function of hepatocytes. It has been observed the sinusoidal deposition of fibrin/fibrinogen and fibronectin in the injured liver and deposition in fibrotic liver tissue during chronic liver injury [40]. Patients with high D-dimer concentration have a higher risk of portal venous thrombosis [41]. The clinical correlation between D-dimer and COLEC10 implies the effect of COLEC10 on modulating the cell interactions of the vascular wall, which is revealed by the RNA sequencing of COLEC10 over-expression in LX-2 cells, shown as Fig. 6F. Therefore, we assume the COLEC10 can be a promising diagnostic marker and prognostic marker of CLD.

To further investigate the in vivo effect of COLEC10 in the pathogenesis of liver fibrosis, overexpression of COLEC10 was mediated via the transduction of AAV with a liver-specific serum type in the CCl4 challenged mice models. The mRNA expression of genes associated with the pathogenesis of liver fibrosis is measured in the COLEC10 overexpression group and vector control group. The results demonstrate the COLEC10 promoted the expression of Col1a1 and Vegfa, which indicates the function of COLEC10 is associated with the ECM alteration and angiogenesis during the liver fibrosis. The in vivo effect of COLEC10 is in accordance with its in vitro effect on LX-2 cells.

In conclusion, the COLEC10 is not produced by hepatocytes but HSCs, especially quiescent HSCs. The expression of COLEC10 is decreased during the progression of liver fibrosis. The COLEC10 is a pluripotent protein with the function of ECM production and alteration, immune response and vascular cell interaction. The serum concentration of COLEC10 is upregulated in the patients with CLD and positively correlated with D-dimer concentration. Collectively, our results identify the producing cell type and the biological functions of COLEC10, highlight the role of COLEC10 in the pathogenesis of liver fibrosis and the promising clinical value as a diagnostic and prognostic markers of CLD. Further studies are needed to fully elucidate the function and clinical significance of COLEC10 in CLD.

### Supplementary information

Supplemetary Figure S1

original data files

checklist

## Data Availability

The raw sequence data of transfected LX-2 cells are deposited in the Genome Sequence Archive in National Genomics Data Center, China National Center for Bioinformation / Beijing Institute of Genomics, Chinese Academy of Sciences (GSA-Human: HRA004962) that are publicly accessible at https://ngdc.cncb.ac.cn/gsa-human. The other datasets including single-cell RNA sequencing data and bulk RNA sequencing data are downloaded from the Gene Expression Omnibus at https://www.ncbi.nlm.nih.gov/geo/. The public available bulk RNA sequencing data used in the manuscript are GSE119606, GSE176042 and GSE193066. The public available data of single-cell RNA sequencing data and single-nuclear RNA sequencing data are GSE206409, GSE212039, and GSE212046.

## Code availability

The analysis of the scRNA-seq data and bulk RNA-seq data was followed by the vignette of Seurat, limma and edgeR using the R software. The authors declared there was no specific unique function used in the analysis.
