# Supplementary material for: The C-type lectin COLEC10 is predominantly produced by hepatic stellate cells and involved in the pathogenesis of liver fibrosis
Source: Cell Death Dis. 2023 Nov 30;14(11):785. doi: 10.1038/s41419-023-06324-8 (PMC10689734; doi:10.1038/s41419-023-06324-8)
Supplement: Supplementary file 1 — Supplemetary Figure S1 [file 41419_2023_6324_MOESM1_ESM.docx]

**Supplementary Figures**

S1. COLEC10 is barely expressed in activated hepatic stellate cells


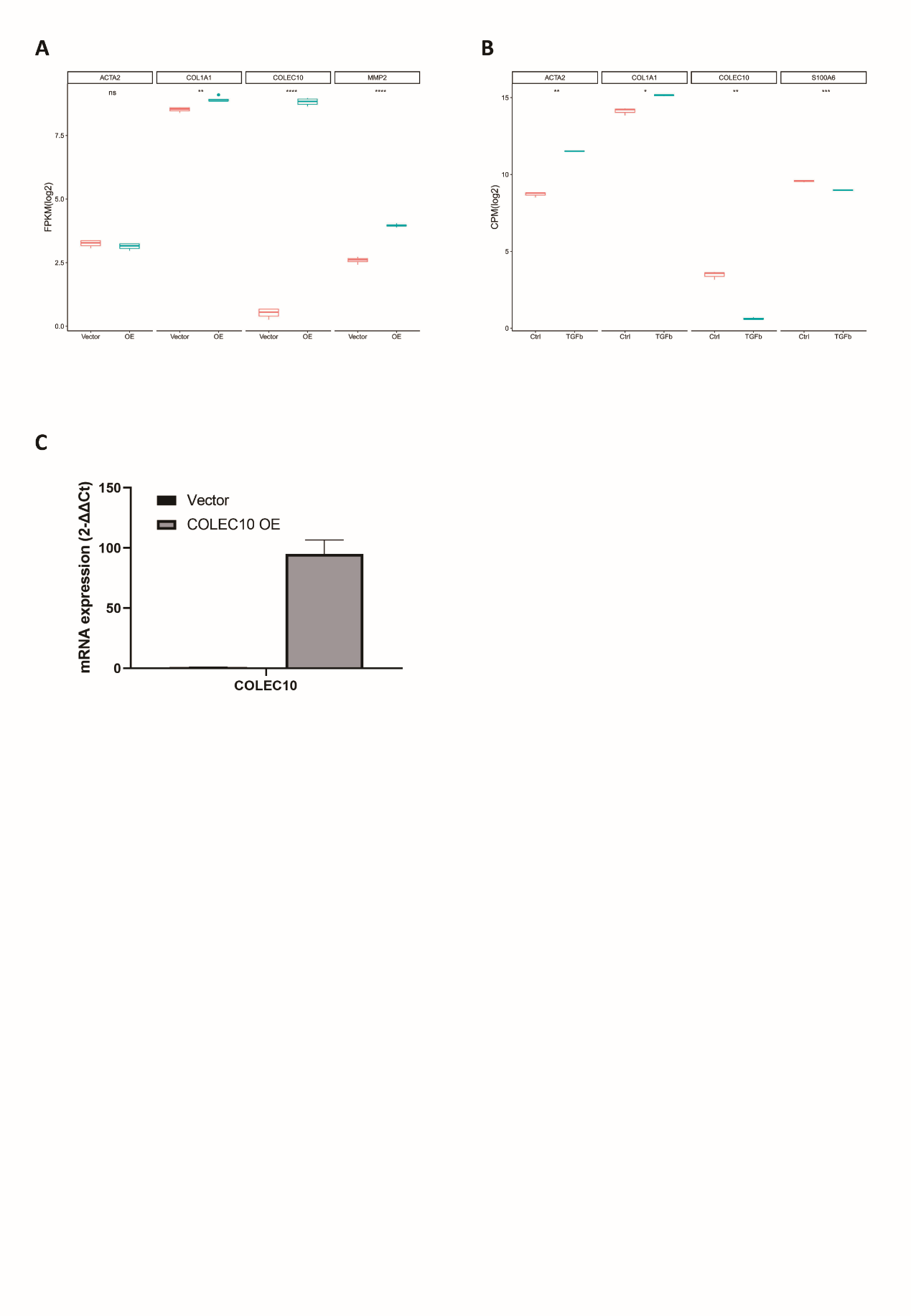


**Figure S1. A** Gene expression profile of RNA sequencing of vector control LX-2 cells and COLEC10 overexpression LX-2 cells. **B** Gene expression profile of RNA sequencing of primary human HSCs treated with or without TGF-β. The data are collected from GEO dataset GSE119606. **C** mRNA expression of COLEC10 in vector control LX-2 cells and COLEC10 overexpression LX-2 cells probed by RT-qPCR.

The RNA sequencing data of vector control LX-2 cells and COLEC10 overexpression LX-2 cells revealed the expression of COLEC10 is quite low in the LX-2 cells, shown as Figure S1A. In addition, the RNA sequencing data of primary human HSCs treated with or without TGF-β revealed the TGF-β inhibits the expression of COLEC10 and increases the expression of ACTA2 and COL1A1, shown as Figure S1B. We probed the mRNA expression of COLEC10 in TGF-β stimulated LX-2 cells and the results showed that the baseline expression of COLEC10 is similar as the water control (data not shown). The mRNA expression of COLEC10 in vector control LX-2 cells and COLEC10 overexpression LX-2 cells was measured with the same primers and the result demonstrates the mRNA expression of COLEC10 was high in the COLEC10 overexpression LX-2 cells, shown as Figure S1C. The results indicated that the COLEC10 is predominantly expressed in the quiescent HSCs.
