# Supplementary material for: The C-type lectin COLEC10 is predominantly produced by hepatic stellate cells and involved in the pathogenesis of liver fibrosis
Source: Cell Death Dis. 2023 Nov 30;14(11):785. doi: 10.1038/s41419-023-06324-8 (PMC10689734; doi:10.1038/s41419-023-06324-8)
Supplement: Supplementary file 2 — original data files [file 41419_2023_6324_MOESM2_ESM.docx]

Figure 6A, expression of COLEC10 in the control and overexpression LX-2 cells


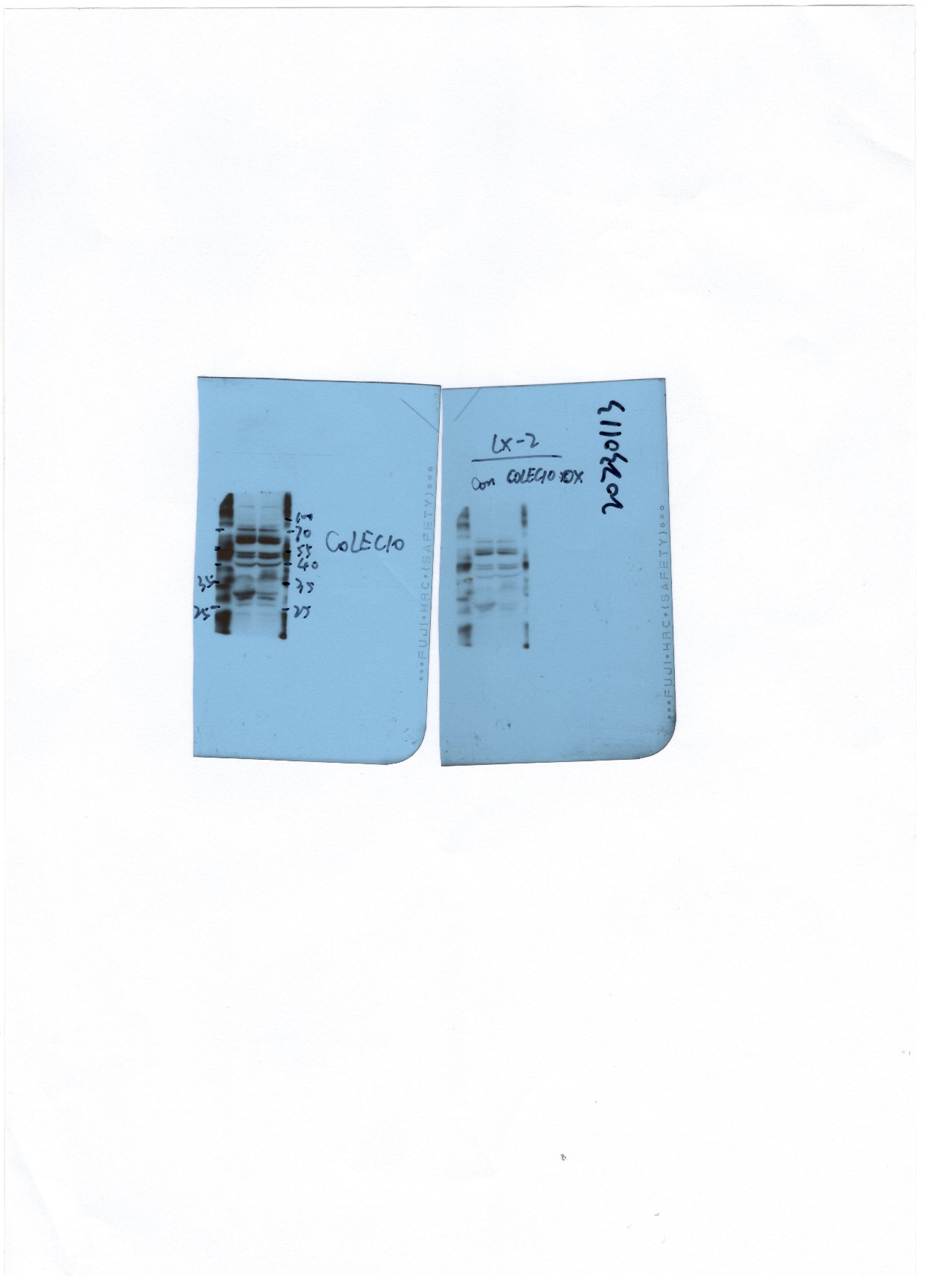


Figure 6A, the expression of loading control in the control and overexpression LX-2 cells


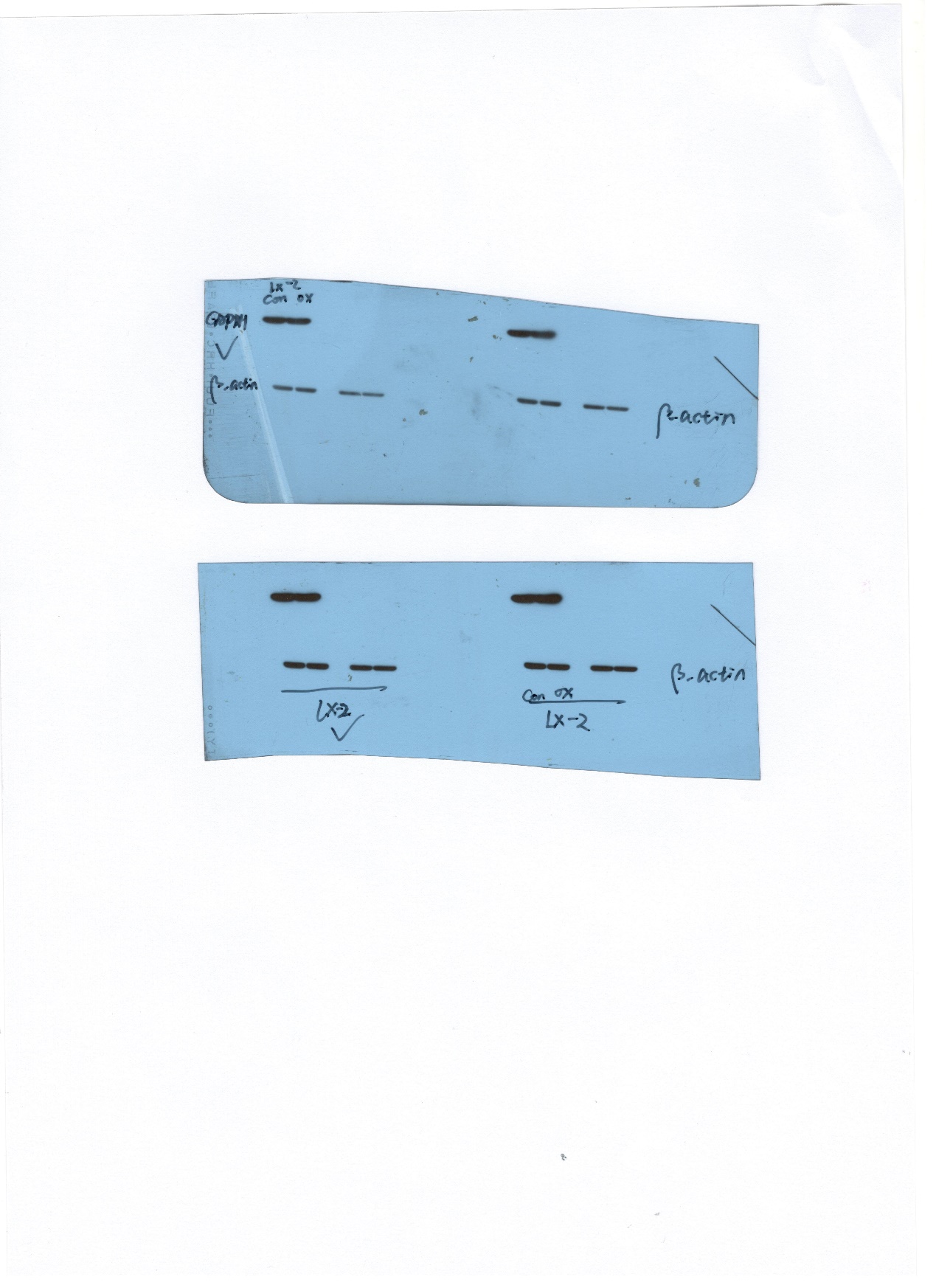


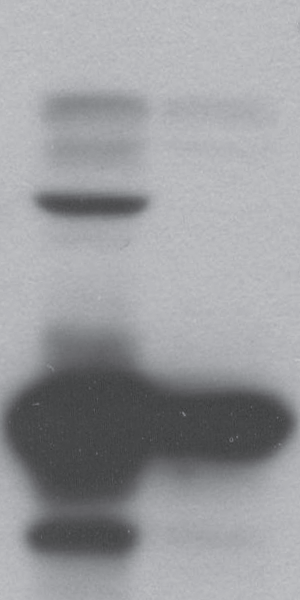
 The expression of GFP in the control and overexpression LX-2 cells.

Figure 7

Expression of the COL1A1 in the control and overexpression LX-2 cells


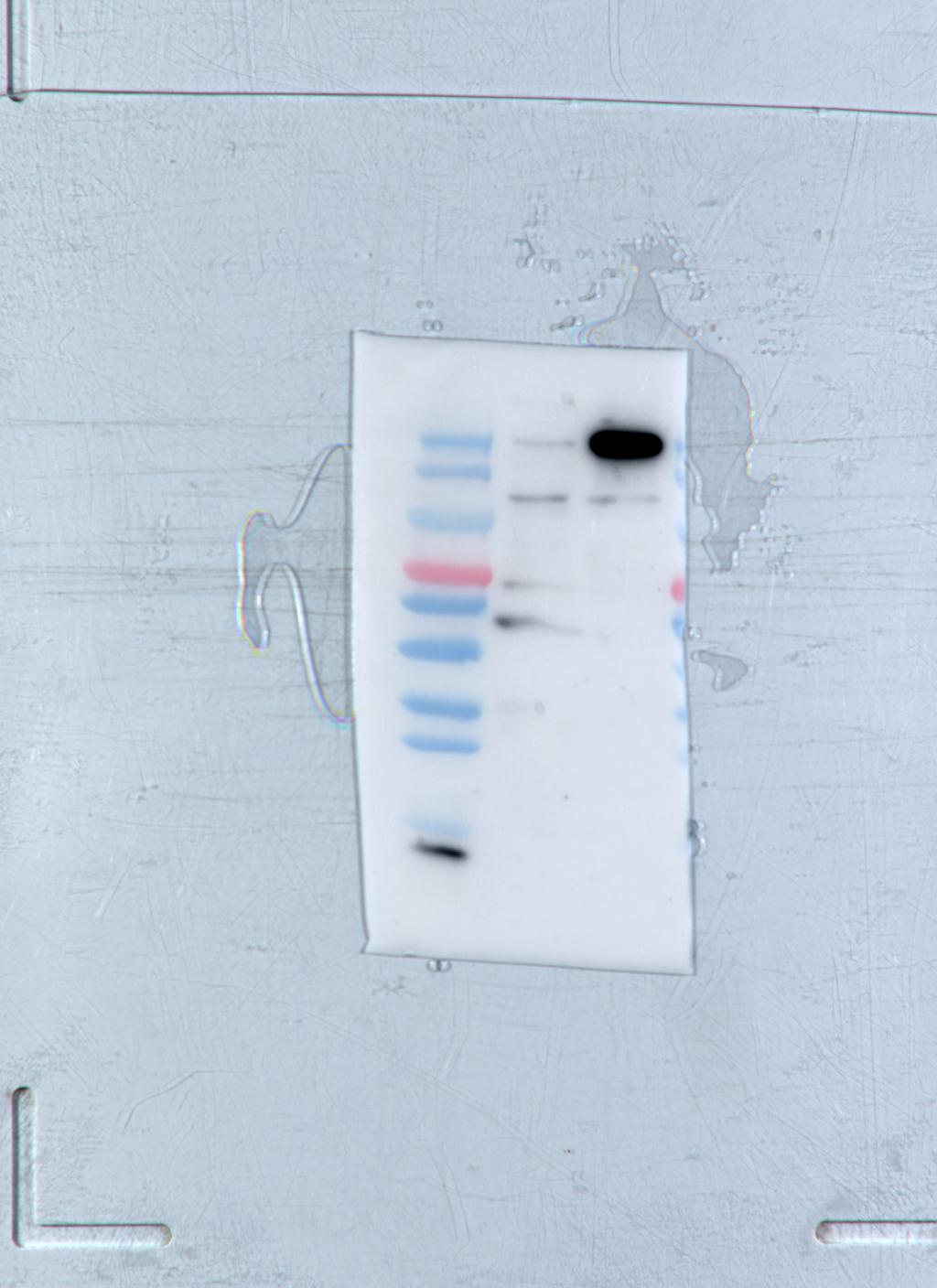

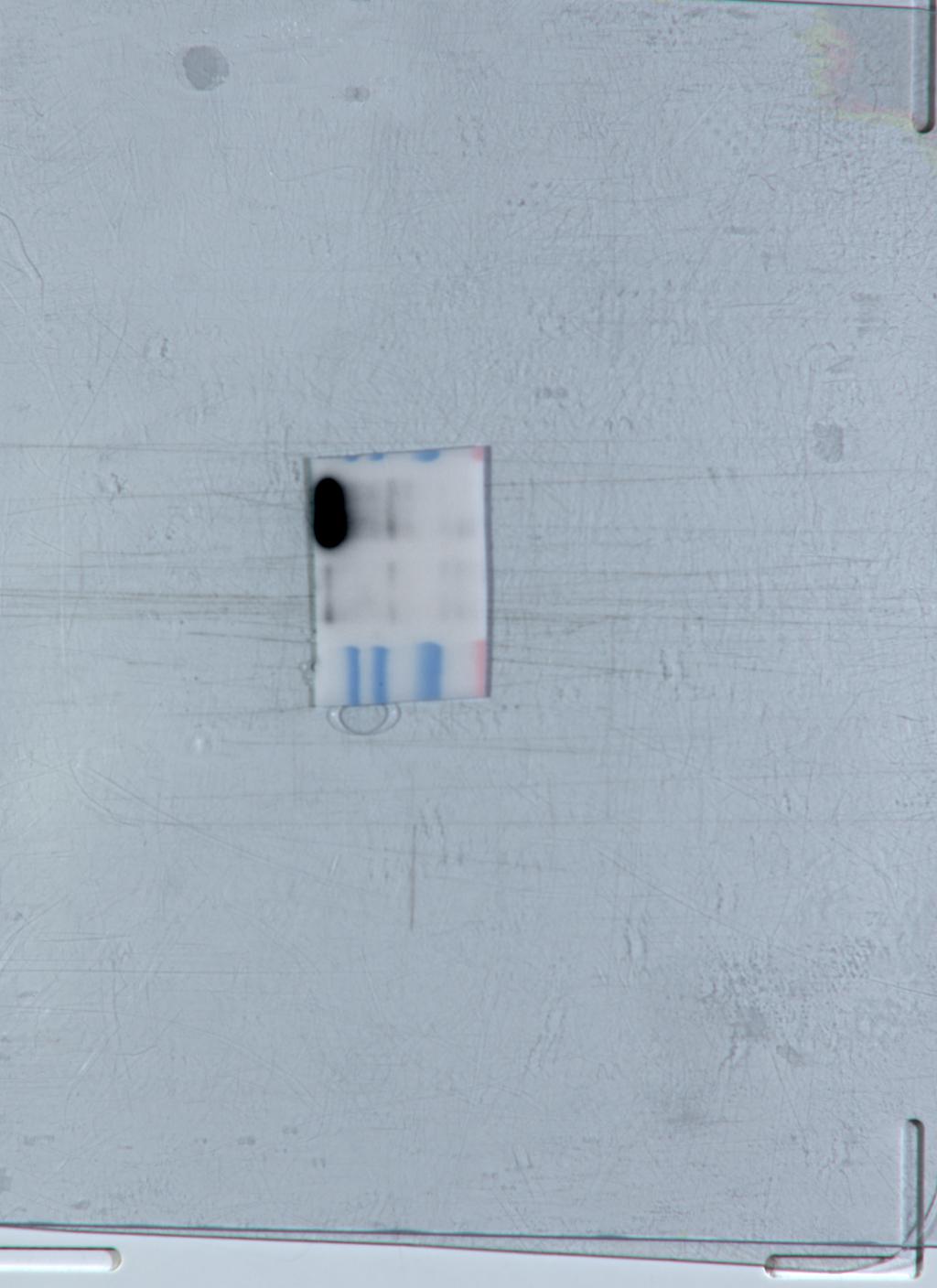


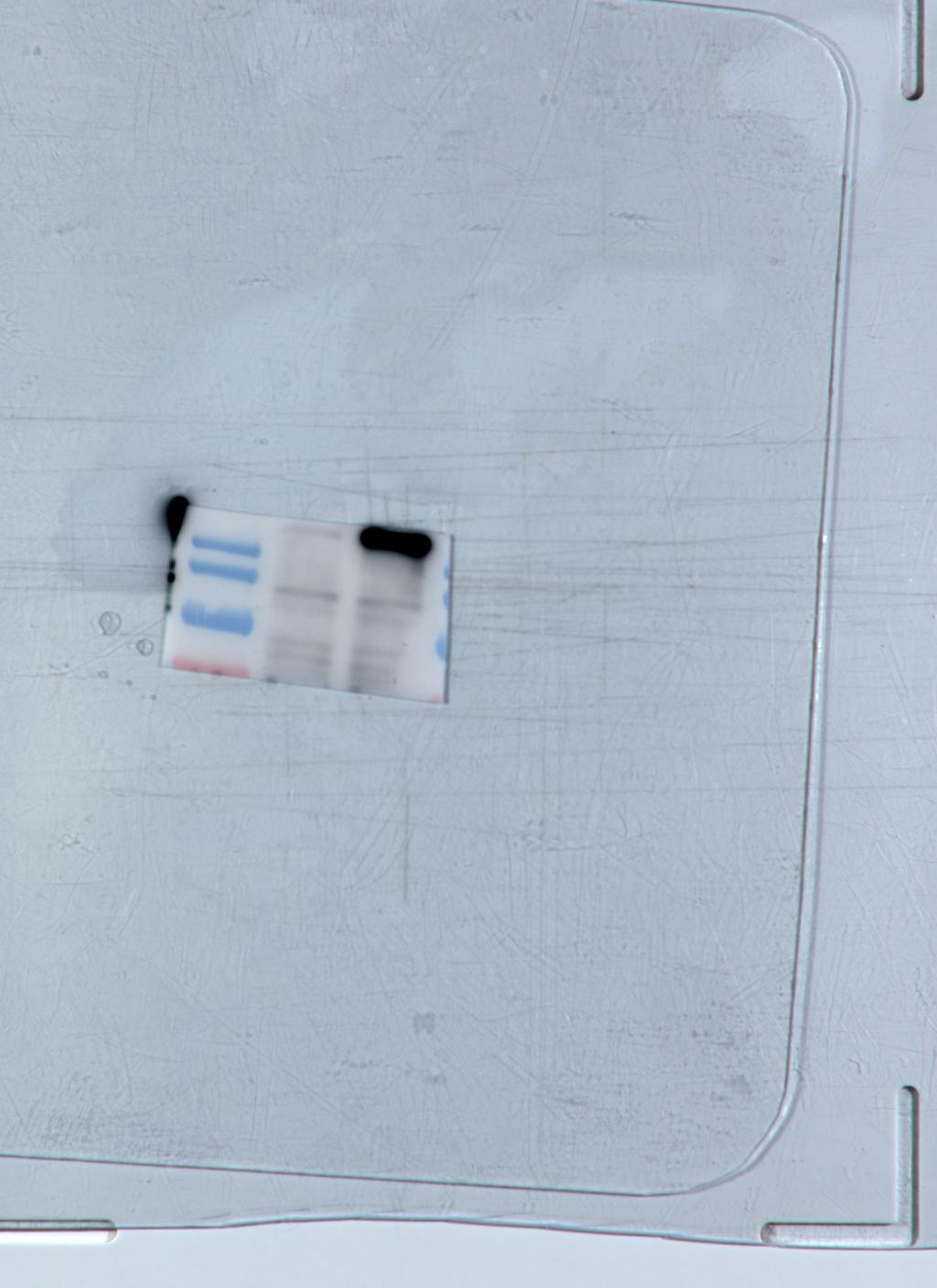


The expression of ACTA2 in the control and overexpression LX-2 cells


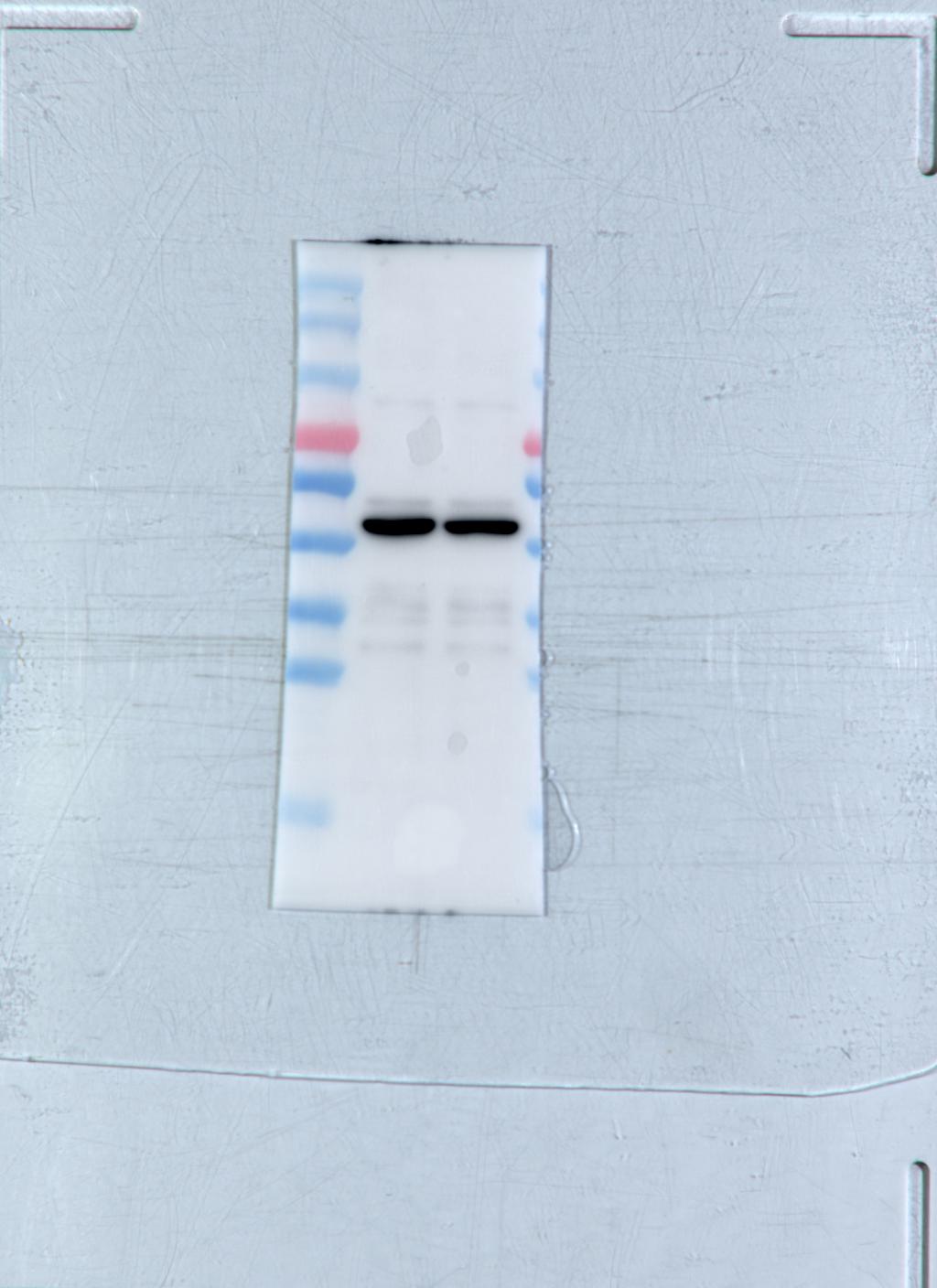

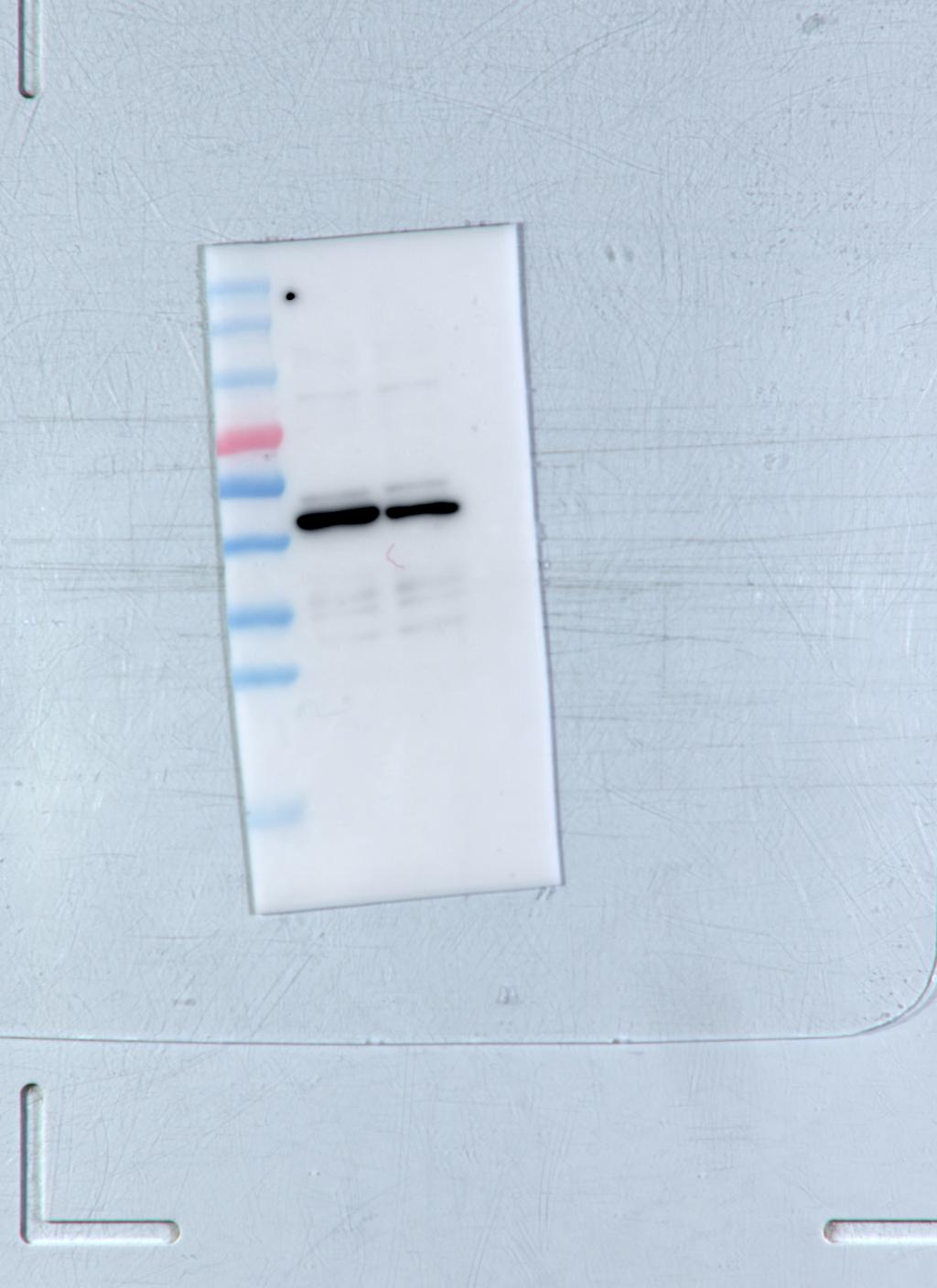

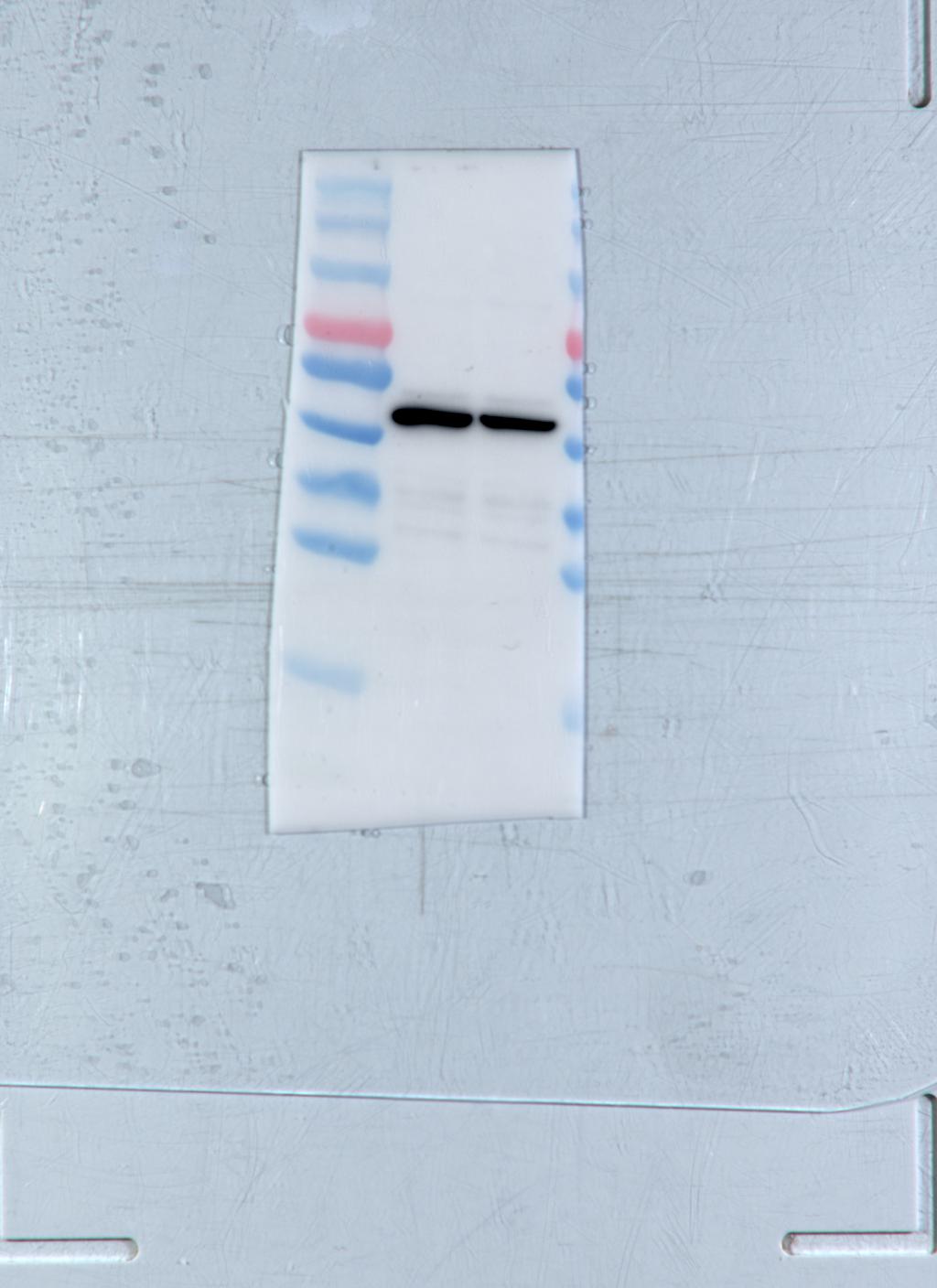


The expression of the beta-actin in the control and overexpression LX-2 cells


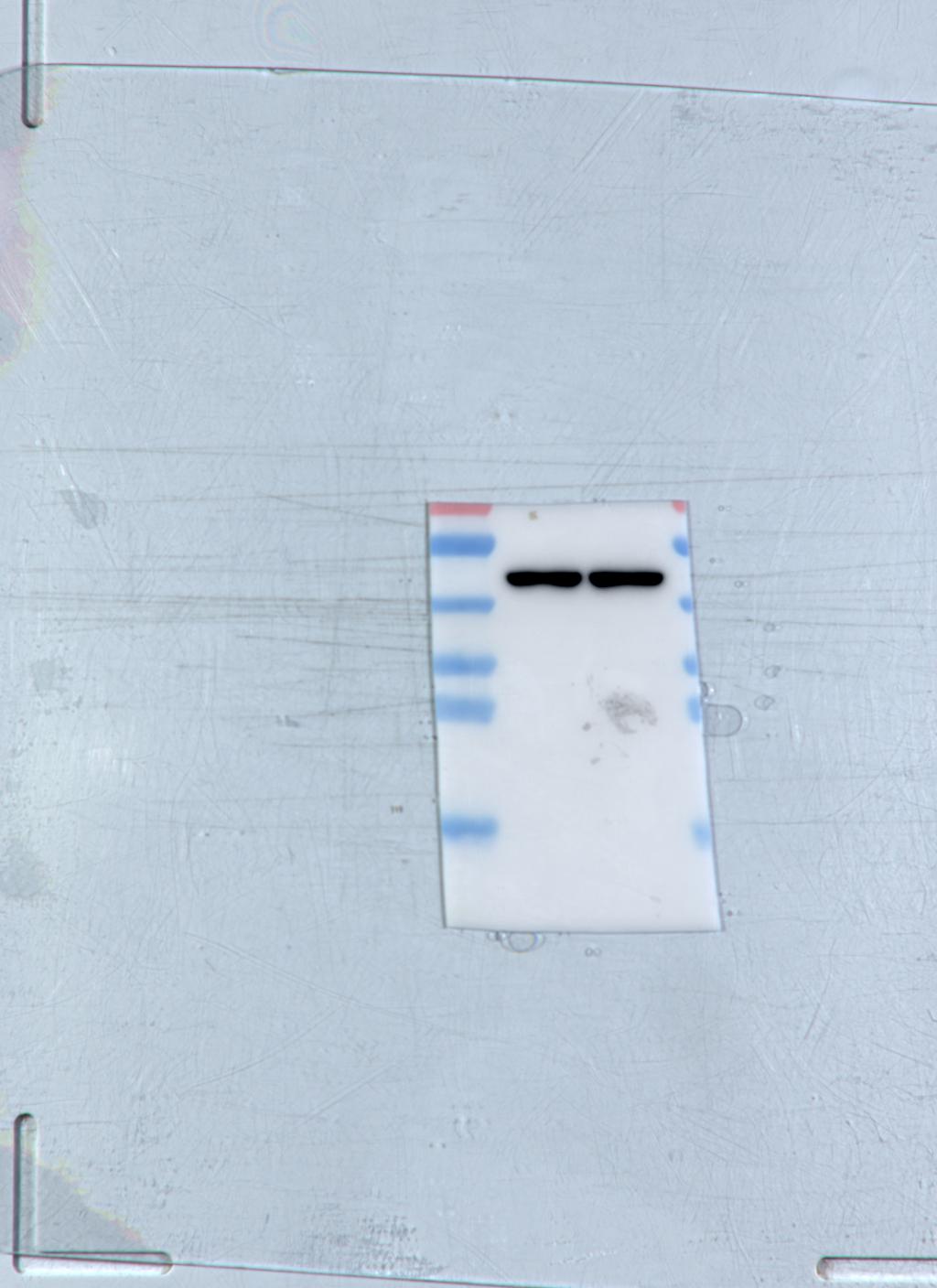

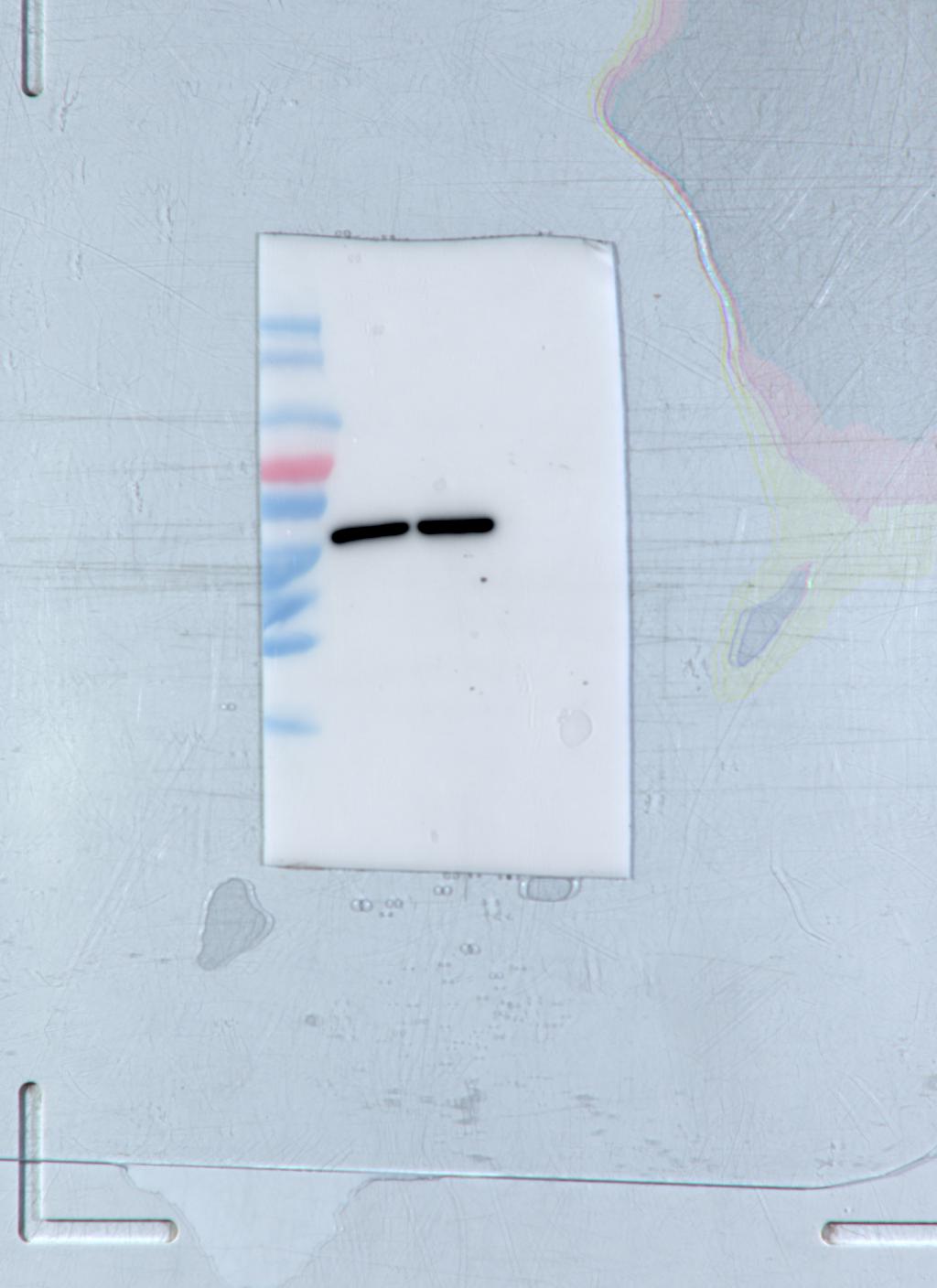

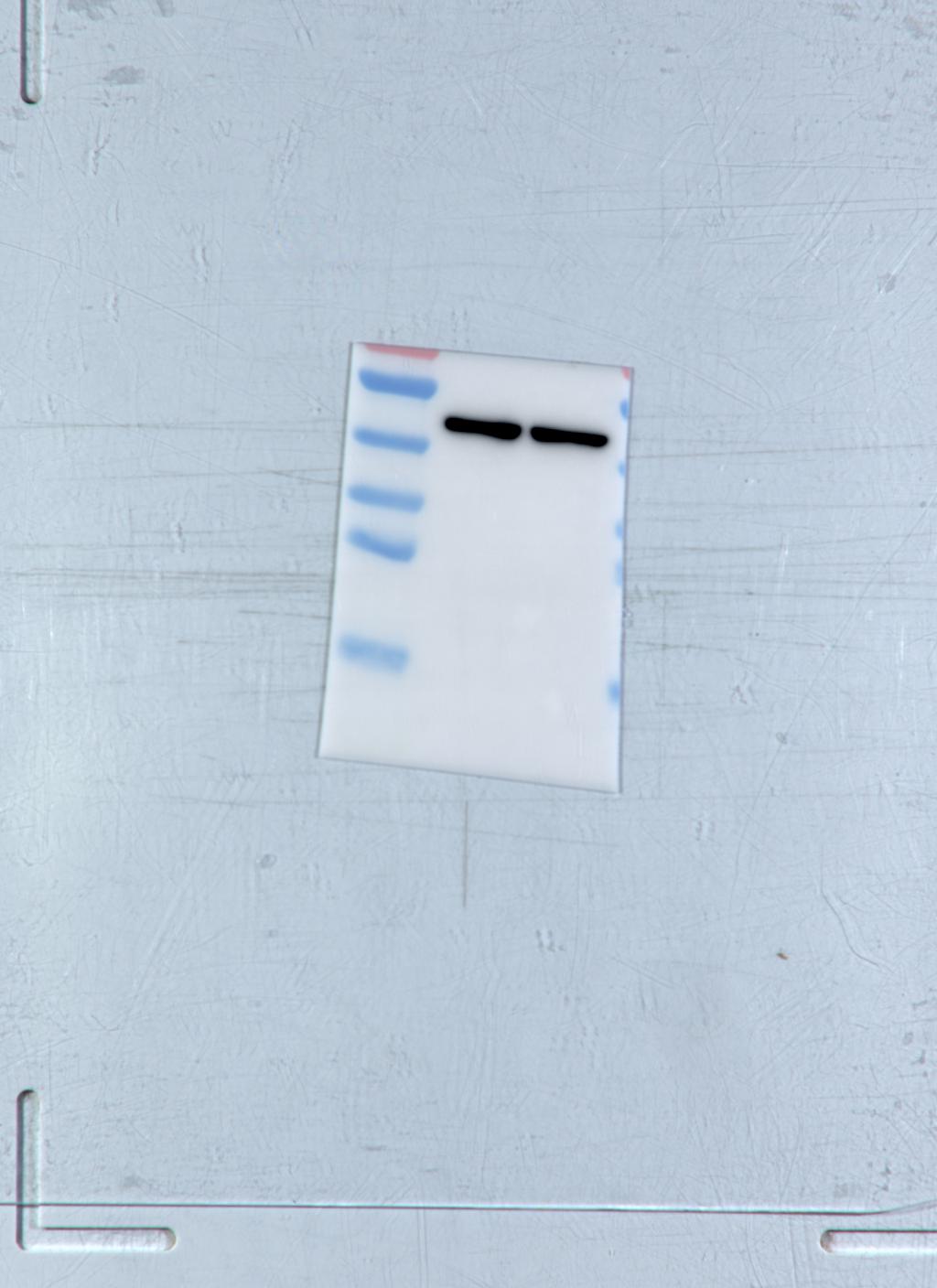


The expression of GAPDH in the control and overexpression LX-2 cells


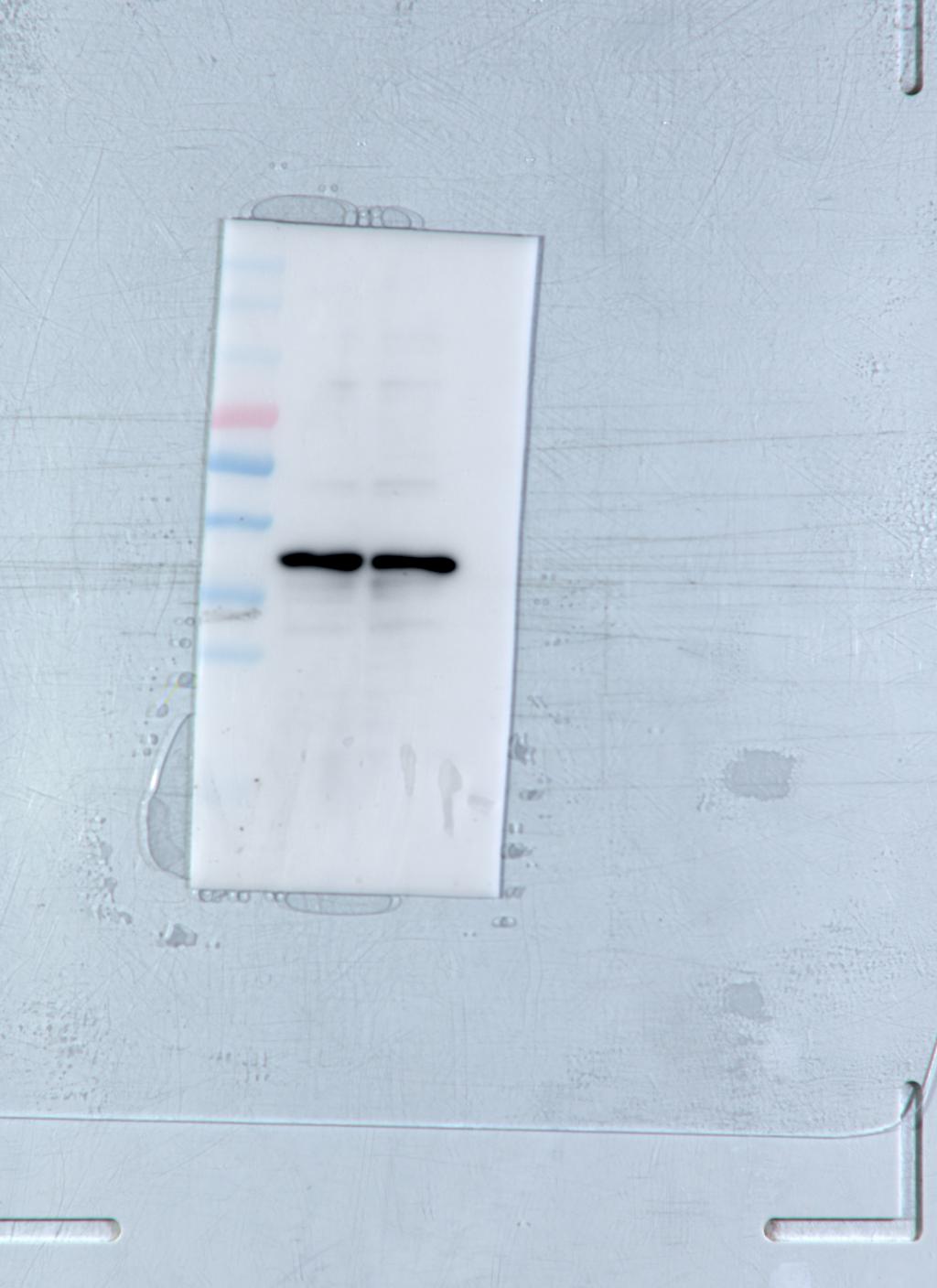


| primary antibody | species | cat num | vendor |
| --- | --- | --- | --- |
| GAPDH | mouse | AF0006 | Beyotime |
| GFP | mouse | AF0159 | Beyotime |
| COLEC10 | rabbit | CSB-PA896556LA01HU | Cusabio |
| COL1A1 | rabbit | PA5-89281 | ThermoFisher |
| COL1A1 | rabbit | AF1840 | Beyotime |
| αSMA | rabbit | 19245 | CST |
| β-Actin | rabbit | 4970S | CST |
| HNF4a | mouse | MA1-199 | ThermoFisher |
| COLEC10 | rabbit | GTX51860 | GeneTex |
| ALB | mouse | AG1078 | Beyotime |
